# Surface oxygenation of multicomponent nanoparticles toward active and stable oxidation catalysts

**DOI:** 10.1038/s41467-020-18017-3

**Published:** 2020-08-21

**Authors:** Shiyao Shan, Jing Li, Yazan Maswadeh, Casey O’Brien, Haval Kareem, Dat T. Tran, Ivan C. Lee, Zhi-Peng Wu, Shan Wang, Shan Yan, Hannah Cronk, Derrick Mott, Lefu Yang, Jin Luo, Valeri Petkov, Chuan-Jian Zhong

**Affiliations:** 1 Department of Chemistry, State University of New York at Binghamton, Binghamton, NY 13902 USA; 2 Department of Physics, Central Michigan University, Mt. Pleasant, MI 48859 USA; 3 CCDC Army Research Laboratory, FCDD-RLS-CC, 2800 Powder Mill Road, Adelphi, MD 20783 USA; 4 School of Materials Science, JAIST, Nomi, Ishikawa 923-1211 Japan; 5 College of Chemistry and Chemical Engineering, Xiamen University, Xiamen, 361005 China; 6 Present Address: College of Engineering, University of Notre Dame, Notre Dame, IN 46556 USA

**Keywords:** Catalysis, Nanoscale materials

## Abstract

The need for active and stable oxidation catalysts is driven by the demands in production of valuable chemicals, remediation of hydrocarbon pollutants and energy sustainability. Traditional approaches focus on oxygen-activating oxides as support which provides the oxygen activation at the catalyst-support peripheral interface. Here we report a new approach to oxidation catalysts for total oxidation of hydrocarbons (e.g., propane) by surface oxygenation of platinum (Pt)-alloyed multicomponent nanoparticles (e.g., platinum-nickel cobalt (Pt–NiCo)). The in-situ/operando time-resolved studies, including high-energy synchrotron X-ray diffraction and diffuse reflectance infrared Fourier transform spectroscopy, demonstrate the formation of oxygenated Pt–NiOCoO surface layer and disordered ternary alloy core. The results reveal largely-irregular oscillatory kinetics associated with the dynamic lattice expansion/shrinking, ordering/disordering, and formation of surface-oxygenated sites and intermediates. The catalytic synergy is responsible for reduction of the oxidation temperature by ~100 °C and the high stability under 800 °C hydrothermal aging in comparison with Pt, and may represent a paradigm shift in the design of self-supported catalysts.

## Abstract (web summary)

Traditional approach to oxidation catalysts relies on metal/oxide supported peripheral interfacial oxygen activity. Here the authors show surface oxygenation of platinum-alloyed multicomponent nanoparticles, and reveals irregular oscillatory kinetics associated with the surface oxygenated sites.

## Introduction

Oxidation reactions, total or partial oxidations, account for more than 60% of the chemicals and intermediates that are widely used in the chemical industry to produce pharmaceuticals, fine chemicals, agricultural chemicals, and various functionalized hydrocarbon molecules, and play a pivotal role in the remediation of hydrocarbon pollutants and the production of sustainable energy^1–6^. Gaseous O_2_ is the most commonly used oxidant, but must often be converted to a more active form or incorporated into an oxide matrix before the oxidation occurs. This requires effective catalysts to activate not only the substrate but also the dioxygen molecule. One example involves low-temperature active and high-temperature stable oxidation catalysts for emission control of critical pollutants such as carbon monoxide, hydrocarbons, and nitric oxides. Traditional approaches seek oxides either as the catalysts or the catalyst supports that are capable of activating, storing, and supplying oxygen, which has been for a long time the basis for the design of oxidation catalysts^7,8^. However, the catalytic performance of such catalysts heavily relies on the oxygen-activation activity at the peripheral interface between the active component and the oxide support, which is not efficient by design and thus poses difficulty in preparing the oxide supported catalysts in order to achieve the full controllability over activity and stability. Traditional low-temperature catalysts for the oxidation reactions that are mostly based on oxides supported platinum or other noble metals^8,9^, exploring the noble metal/support perimeter zones^6^ in nano oxide/micro metal interface^10^, isolated single atoms^3,11,12^, reactive surface intermediates^13^, surface oxidized species^14^, metal carbonylation^15^, meso/nano porous structure^16^, and the lattice oxygen^17^. While insights into these surface sites have been gained by spatial and time-resolved techniques^18–22^, the instability of the surface sites on such supported catalysts due to Pt mobility for particle sintering, coke formation, and PtO formation poses a major challenge^19–26^.

Here, we show a new approach to the design of the oxidation catalysts by surface oxygenation of a multicomponent alloy of Pt with oxyphilic metals such as Ni and Co under catalytic reaction condition. The surface oxygenation of the multimetallic alloy nanoparticles creates oxygenated active sites on the catalyst, which differs from traditional metal/metal oxide support catalysts where the catalytic reaction occurs on metal/support perimeter zone^6–13^. The ternary catalyst design features multifunctional surface active sites with active Pt center and self-generated and self-perished surface oxygen-activating NiO/CoO sites, enabling not only composition-controllable but also dynamically tunable activity and stability since the degree of surface oxygenation depends on the alloying composition and phase structures. The catalyst of (PtNiCo)^alloyed^(PtNiOCoO)^surface-oxygenated^ is demonstrated by supporting it on oxygen-inactive Al_2_O_3_ support for the total oxidation of propane as a model system. With the design of the surface-oxygenated multimetallic alloy catalysts, the dynamic nanocrystal and surface structures of the catalysts were probed under the reaction condition for total oxidation of propane using a combination of in situ/operando techniques, including high-energy synchrotron X-ray diffraction and diffuse reflectance infrared Fourier transform spectroscopy (DRIFTs). The dynamically controllable surface oxygenation and catalytic synergy are evidenced by the lattice expansion/shrinking, ordering/disordering, metal-oxygen coordination^27^, and surface intermediates formation kinetics in correlation with reaction kinetics of reactants and products. The capture of the “regular–irregular oscillatory kinetics” provides a new insight into the remarkable catalytic activity and stability of the catalyst structure with an alloy core and a surface-oxygenated layer, i.e., NA^core^–SONA^surface^, or simply NA–SONA. This is to our knowledge the first example of this type of catalysts and may represent a paradigm shift toward achieving self-supported active and stable catalysts by design.

## Results

### Morphology and structure of surface-oxygenated catalysts

The precursor nanoparticles were prepared by synthesizing first the alloy nanoparticles with controlled ternary composition (Pt_*n*_Ni_*m*_Co_100−*n*−*m*_) (*n* + *m* < 100) in a wet-chemical reduction reaction. The precursor nanoparticles were then supported on alumina. For example, the as-synthesized ternary (*n* = 42, *m* = 39) alloy nanoparticles feature an average size of 4.9 (±0.6) nm and a lattice spacing of 0.188 nm characteristic of (111) crystal plane (Supplementary Fig. 1a, b). Aberration corrected high-angle annular dark-field (HAADF)-scanning transmission electron microscopy (STEM) image and fast Fourier transform patterns of single nanoparticle oriented at <110> zone axis (Supplementary Fig. 2a, bottom) reveal streaks in [111], [002], and [022] directions, whereas only [002] and [022] directions occur in <100> zone axis (30° tilted, Supplementary Fig. 2a, top), featuring a truncated octahedron structure (Supplementary Fig. 2b, c). A close examination of the intensity oscillation pattern between bright (Pt) and dark (Ni, Co) and the lattice spacing from the HAADF-STEM data (Supplementary Fig. 2b) further confirms the presence of Ni and Co atoms in the nearest neighbor of Pt atoms.

**Fig. 1:**
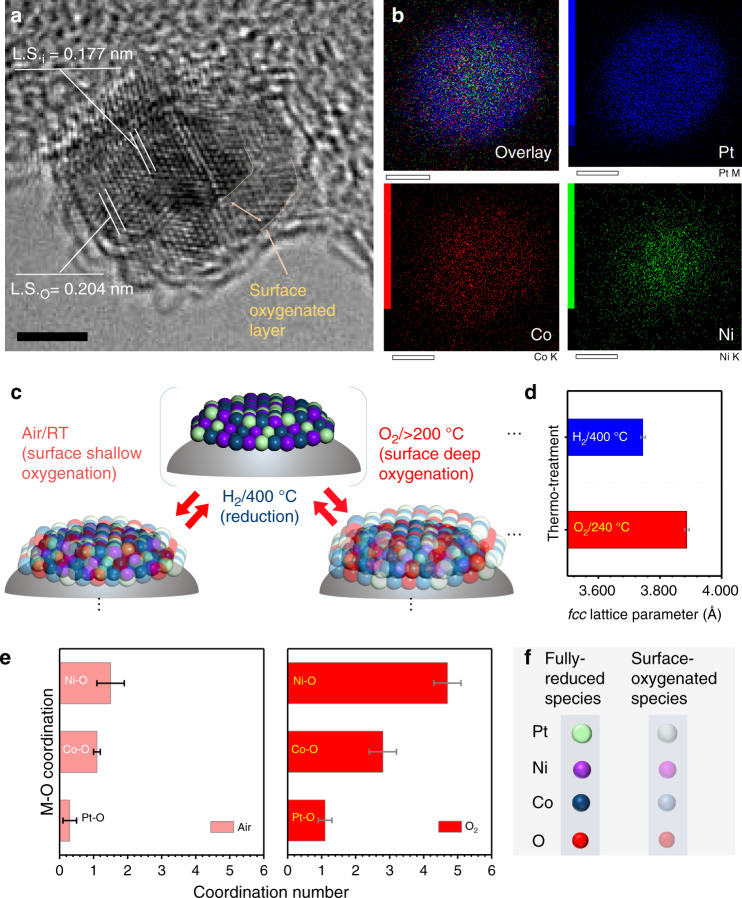
Morphology and structure of (PtNiCo)^core^(PtNiOCoO)^surface^. **a** HR-TEM of PtNiCo–PtNiOCoO/Al_2_O_3_ (*n* = 42, *m* = 39, 1.0 wt%), showing lattice spacing at edge (noted L.S._O_, 0.204 nm); and lattice spacing in the center (noted L.S._i_, 0.177 nm); scale bar = 2.0 nm; **b** the corresponding EDX elemental mapping, Pt (blue), Ni (green), and Co (red) in **a**. **c** illustration of the fully reduced ternary alloy state and the surface-oxygenated states of two different oxygenation degrees under the indicated conditions; **d** the fcc lattice parameter change corresponding to the reduced and oxygenated states as determined by in situ HE-XRD/PDF analysis; **e** the M–O coordination number corresponding to the surface-oxygenated states as determined by EXAFS spectral analysis; and **f** the symbols and legends corresponding to the illustration in **c**.

Key to the catalyst preparation is the surface oxygenation, which involves thermochemical oxidation under controlled oxygen atmosphere, producing (Pt_*n*_Ni_*m*_Co_100−*n*−*m*_)^core^(PtNiOCoO)^surface^ (*n* + *m* < 100). We examined next the morphology of the same nanoparticles supported on alumina after the surface oxygenation by the thermochemical oxidation. The catalyst showed a lattice spacing of 0.204 nm at edges and 0.177 nm in the center (Fig. 1a), indicative of a surface layer of PtNiOCoO with ~1.4 nm thickness which accounts for 6–7 atomic layers.

By synchrotron high-energy X-ray diffraction coupled to pair distribution function analysis (HE-XRD/PDFs) analysis of NA–SONA catalysts (Supplementary Fig. 3), the normalized compressive strain Δα/α (vs. Pt) in terms of lattice parameter (L.P.) is found to be ~0.8% for PtNiCo–PtNiOCoO/Al_2_O_3_ (*n* = 45, *m* = 33) (L.P. ~ 3.886 Å) and ~3.2% for carbon black-supported catalysts (L.P. ~ 3.792 Å), suggesting limited lattice  shrinking for the former. The compressive lattice strain of PtNiCo with respect to Pt is due to lattice shrinking by alloying with the transition metals, the degree of which reflects the insertion of oxygen in the lattice, and a subtle deviation from Vegard’s law, depending on the degree of oxidation. The former also showed a “satellite” peak ~2.125 Å, which is characteristic of a limited surface oxidation. While there is an indication of slight Ni enrichment in the core (Fig. 1b) consistent of shorter lattice spacing in the center (Fig. 1a), the data of EELS line profiles show that Pt, Ni, and Co species are uniformly distributed across the entire NPs, confirming their alloy-type character (Supplementary Fig. 4). Similar surface lattice expansion on carbon black-supported PtNiCo–PtNiOCoO catalysts was also observed (Supplementary Fig. 5). The oxygenation and reduction of the catalysts (Fig. 1c) were analyzed by extended X-ray absorption fine structure (EXAFS) and HE-XRD/PDF analysis (Fig. 1c–f). By analyzing the L.P.s based on the HE-XRD/PDF data (Fig. 1d) and the coordination number (CN) of metal components based on EXAFS spectral fitting (Fig. 1e), the catalysts were found to feature a long range disordered alloy character in the core with different degrees of metal oxygenation. The detection of the high level of oxygenation for Ni and Co (N(Ni–O) ~ 4.7, N(Co–O) ~ 2.8) and the low level of Pt oxygenation (N(Pt–O) ~ 1.1) reflects the ability to harnessing the surface oxygenation. It is important to note that the pre-alloyed composition was basically preserved even by thermal treatment at a temperature as high as 750 °C. In comparison with the PtNi and PtCo catalysts, a higher degree of surface oxygenation was found for PtNiCo catalysts after the reductive treatment followed by exposure to ambient condition (see additional EXAFS and HE-XRD characterizations in Supplementary Tables 1 and 2).

### Catalytic activity and stability

**Fig. 2:**
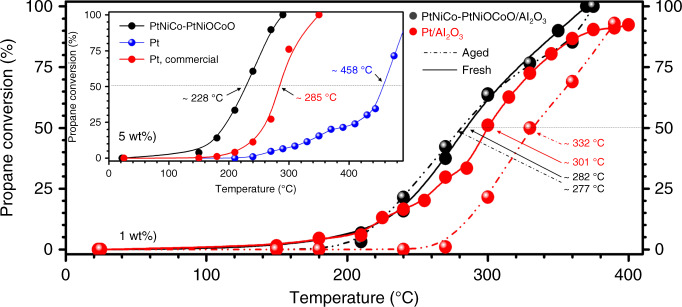
Propane oxidation over (PtNiCo)^core^(PtNiOCoO)^surface^ catalyst. Propane conversion over the ternary catalyst derived from PtNiCo–PtNiOCoO/Al_2_O_3_ (*n* = 42, *m* = 39, 1.0 wt%, black) and Pt/Al_2_O_3_ (1.0 wt%, red): freshly prepared (solid curve), and hydrothermally aged under 10% CO_2_ + 10% H_2_O + N_2_ at 800 °C for 16 h (dash curve). The values of *T*_50_ (i.e., the temperature at which 50% conversion is achieved) are indicated in the plots. Inset: propane conversion of PtNiCo–PtNiOCoO/Al_2_O_3_ (black, 5.0 wt%) in comparison with commercial Pt/Al_2_O_3_ (red, 5.0 wt%) and Pt/Al_2_O_3_ prepared by the same method (blue, 5.0 wt%). Reactor: quartz tube microreactor packed with catalyst fixed bed at a flow rate of 50 ml min^−1^. The standard deviation is ±0.3%.

The high catalytic activity for the PtNiCo–PtNiOCoO/Al_2_O_3_ catalyst (*n* = 42, *m* = 39, 1.0 wt% total metal loading) in propane oxidation was evidenced by the fact that *T*_50_ ~ 282 °C (Fig. 2) was lower than that of commercial Pt/Al_2_O_3_ catalyst with the same total metal loading (*T*_50_ ~ 301 °C). The difference was much greater when comparing the catalysts with 5.0 wt% metal loading, e.g., *T*_50_ ~ 228 °C for PtNiCo–PtNiOCoO/Al_2_O_3_ and ~285 °C for commercial Pt/Al_2_O_3_, and ~458 °C for Pt/Al_2_O_3_ prepared by the same method as for the PtNiCo–PtNiOCoO/Al_2_O_3_, Fig. 2 inset). The NA–SONA features superior stability, showing no indication of deactivation after 800 °C hydrothermal aging (*T*_50_ ~ 277 °C, 1.0 wt%). For Pt_*n*_Ni_*m*_Co_100−*n*−*m*_–PtNiOCoO/Al_2_O_3_ (*n* = 42, *m* = 39), the composition of the aged catalyst remained basically unchanged. For Pt/Al_2_O_3_ catalyst (Fig. 2), the decay of catalytic activity is evidenced by an increase of *T*_50_ to 332 °C after the 800 °C hydrothermal aging.

### In situ/operando studies of the dynamic structures

**Fig. 3:**
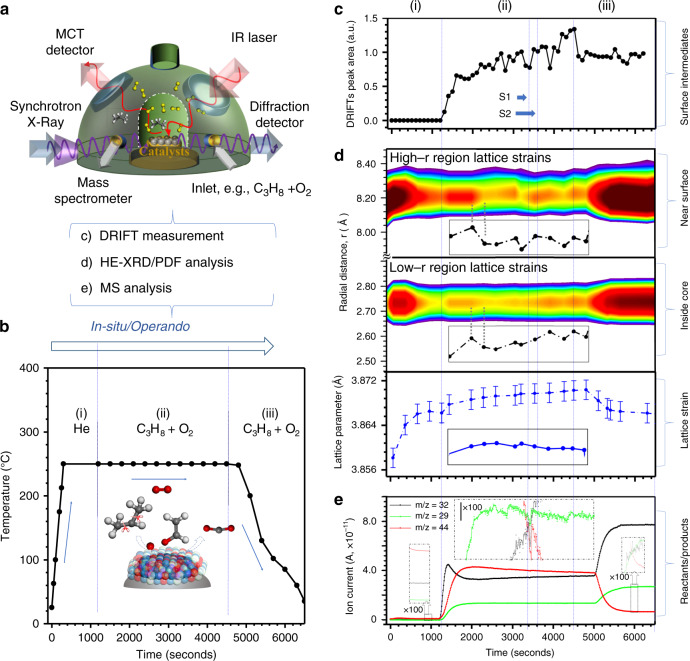
In situ/operando DRIFTS, HE-XRD/PDF, and MS characterizations. **a** The in situ/operando measurement setup; **b** the reaction conditions (Inset illustrates reactant, intermediate, and product over the oxygenated catalyst surface); **c** plot of integrated peak area in 1300–1800 cm^−1^ as a function of time (black curve) from in situ DRIFTS spectra (catalyst loading 5.0 wt%) operated at 250 °C for 1 h followed by cooling back to RT for 30 min; major intermediates identified: (1) 1732 cm^−1^ (linear adsorption of aliphatic ester (R–C(=O)O–)); (2) 1650–1645 cm^−1^ (bridge adsorption of bicarbonate (HOCOO^−^) or stretching mode of adsorbed H_2_O); (3) 1590–1576 cm^−1^ (υ_as_(R–COO^−^)), 1458 cm^−1^ (υ_s_(R–COO^−^)), and 1376 cm^−1^ (*δ*_CH_) linked to bridge adsorption of acetate (CH_3_COO^−^) or formate (HCOO^−^); (4) 1626–1615 cm^−1^ (υ_as_), 1398 cm^−1^ (υ_s_), and 1337 cm^−1^ (*δ*_CH_) (atop adsorption of enolate (CH_2_=CHO^−^)); and (5) 1470 cm^−1^ (υ_as_ (CH_3_–O^−^)) and linear adsorption of methoxy species (CH_3_–O^−^)). The relative standard deviation (RSD) is 0.7%. **d** Comparison of the in situ data of lattice parameter (blue curve); and the Pt–Pt bonding distance at 2.747 Å (low-*r*) and 8.210 Å (high-*r*) regions; **e** analysis of *m*/*z* = 29, 32, and 44 characteristic of C_2_H_5_^+^, O_2_^+^, and CO_2_^+^ as a function of time, Inset: Magnified views for *m*/*z* = 29, 32, and 44 fragments by a factor of ×100. Note that atomic-level changes in interatomic distances are more significant with the higher-*r* PDF peaks at 8.210 Å than the lower-*r* PDF peaks at 2.675 Å. Note that stage 1 (S1) indicates a depletion of surface intermediates along with a structural disordering and lattice expansion. Stage 2 (S2) indicates a growth of surface intermediates along with structural ordering. The 3D plots of the full spectra of DRIFTS, HE-XRD/PDF, and the MS data are shown in Supplementary Fig. 6.

Under propane oxidation reaction condition, we performed a time-resolved study using combined in situ HE-XRD/PDFs, DRIFTs, and online mass spectroscopy (MS) (Fig. 3a, b). Recent spatial and temporal characterizations of other systems have indicated the possibilities of these techniques^28^ to identify “soft” and “hard” atomic-scale structural disordering under CO oxidation reaction^29^. Operando DRIFTs data over Pt_*n*_Ni_*m*_Co_100−*n*−*m*_–PtNiOCoO/Al_2_O_3_ catalyst (*n* = 42, *m* = 39, 1.0 wt%) (Fig. 3c) were recorded every 100 s in the following sequence (Supplementary Fig. 6a): (i) heating under He, (ii) propane oxidation at 250 °C for 1 h, and (iii) cooling down to room temperature (RT). The results reveal five types of major species at different frequency regions (I–V, Supplementary Fig. 7a). Types II–V species were detected over the catalyst under (ii) isothermal process (Fig. 3c), with the peak absorbance being proportional to intermediates’ concentration and showing an oscillatory pattern (with a period of ~700 s), reflecting a dynamic growth and removal of surface oxycarbon intermediates (Fig. 3d). During cooling (iii), new peaks appear at 1546, 1436, and 1425 cm^−1^ (propionate species) below 200 °C, suggesting a low activity for C–C cleavage (Fig. 3c). In contrast, Type II–V species were not detected for Pt catalyst, but Type I at 1727 (aliphatic ester, υ_as_(RC(=O)O)) and 1701 cm^−1^ (acetone, υ_as_((CH_2_)_2_C(=O))) were detected (Supplementary Fig. 7b), which were less reactive and typically more strongly bonded on the surface^24^.

Simultaneously with DRIFTs measurement, the HE-XRD/PDFs patterns (Supplementary Fig. 6b) were obtained every 300 s. The NA–SONA features an fcc alloy structure characteristic during entire process. During (i), heating under He atmosphere, the atomic structure of the catalyst underwent a moderate relaxation with a small degree of thermal expansion, leading to a uniform increase in Pt–Pt bonding distances from 2.728 Å at RT to 2.734 Å at 250 °C (Fig. 3d and Supplementary Figs. 7b and 8). The PDF peaks, including first Pt–Pt bonding distance, showed no extra broadening, indicating negligible changes in the degree of local structural disorder. Upon further exposure to C_3_H_8_ + O_2_ atmosphere (ii), the catalyst exhibits both structural expansion and increased local structural disorder as evidenced by the concurrent peak shift, broadening, and intensity decrease of PDF peaks. The atomic structural disordering/ordering and structural expansion/shrinking (Fig. 3d) follow an oscillatory pattern (with a period of ~700–1000 s). This oscillation is concomitant with a continuous increase of the fcc L.P. from 3.866 Å at RT to 3.870 Å at 250 °C, and the growth/removal of oxycarbon surface intermediates as observed in DRIFTs (Fig. 3c). The subsequent cooling (iii) resulted in a shift of PDF peaks to lower *r* values, e.g., the first Pt–Pt bond distance showing a partial relaxation to 2.734 Å at RT from 2.737 Å at 250 °C and increased ordering mainly due to the temperature drop. Note that the shoulder peak of the first PDF peak at 2.130 Å, characteristic of the presence of a limited surface oxidation, remains unchanged (Supplementary Fig. 8). This process was also followed by simultaneous analysis of the tail gas composition by mass spectrometry (Supplementary Fig. 6c). The *m*/*z* = 29, 32, and 44, features, corresponding to reactants C_3_H_8_ and O_2_ and product CO_2_, respectively, were found to display a “breathing pattern” (interval of 700 s) for the surface-oxygenated catalyst (Fig. 3e). The concurrent negative spikes suggest that the oscillatory pattern stems from the change in partial pressure due to a sudden depletion of intermediates, observed in DRIFTs spectra (Fig. 3c). In the gradual increase of O_2_, the initial spike is indicative of the role of surface and lattice active oxygen in the catalytic reaction. This process was accompanied by the decrease of CO_2_ and the plateau of C_3_H_8_, indicating that the surface intermediates also played a role in regulating the catalytic reactivity. This finding is suggestive of some correlation in terms of the dynamic evolution of the surface species (Fig. 3c) and the corresponding structural disordering/ordering (Fig. 3d). Note that the apparent catalytic activity on propane stream from the results of the in-house testing and the in situ mass spectroscopic measurement shows a steady curve with subtle fluctuations (Supplementary Figs. 9 and 10). After ~500 s delay in structural evolution, the structural change is dominant in the catalytic behavior. In contrast, the data for propane oxidation over Pt catalyst showed only a slow and large increase of the fragment (Supplementary Fig. 10), indicative of a graduate surface poisoning as observed in DRIFTs data (Supplementary Fig. 7b).

By comparing the surface intermediates formation, the L.P. and Pt–Pt coordination evolution and the C_2_H_5_^+^ fragment evolution in real time (Fig. 3c–e), a major finding is that the reaction over the catalyst approaches a steady state in the initial 600 s via rapid consumption of propane, fast growth of surface intermediates, and quick lattice expansion, all occurring at a relatively similar speed. Secondly, the surface intermediate species gradually fall to a “valley” in the “breathing pattern” in which the catalyst displays a certain degree of disordering. The oscillatory depletion of surface-oxygenated intermediate species played an important role in introducing lattice oxygen and structural disordering on the NA–SONA catalyst (Fig. 3d). The role played by the lattice oxygen was further substantiated by monitoring propane only stream over the oxygenated catalysts (Supplementary Fig. 11), where the decrease of propane and the increase of CO_2_ for the oxygenated ternary alloy catalysts are in sharp contrast to the data for the Pt catalyst.

### Tunability of the catalytic properties

The catalytic activity of propane oxidation over the catalysts with similar sizes but different ternary compositions, e.g., *n* = 22 and *m* = 29 (Supplementary Fig. 12a, c), and *n* = 81 and *m* = 1 (Supplementary Fig. 12b, d), and their binary counterparts, showed subtle composition dependence (Supplementary Fig. 13), and dependence on supports, thermochemical treatment conditions and total metal loading (Supplementary Fig. 14). These results are clearly supportive of a surface oxygenation related catalytic activity enhancement and tunability. Importantly, the alloying characteristics were shown to sustain even at the high temperature (Supplementary Fig. 15). Analysis of the kinetic parameters (e.g., activation energy (*E*_*a*_), Pt-specific reaction rate (*R*) and Pt-specific turnover frequency (TOF)) was performed (Supplementary Table 3). This estimate is reasonable since Pt remains on the surface as part of the surface-oxygenated layer, and the non-noble metal species function as an oxygen reservoir for oxygen activation. The results showed a higher *R* (5.9 × 10^−4^ mol/(g_Pt_ s)) and a greater TOF (1.2–1.5 s^−1^) over PtNiCo–PtNiOCoO/Al_2_O_3_ (*n* = 42, *m* = 39) than those of commercial Pt/Al_2_O_3_ catalyst (3.2 × 10^−4^ mol/(g_Pt_ s), 0.46 s^−1^). These findings support that the catalytic activity originates from the ternary compositions.

### Identification of surface intermediates of oxidation

**Fig. 4:**
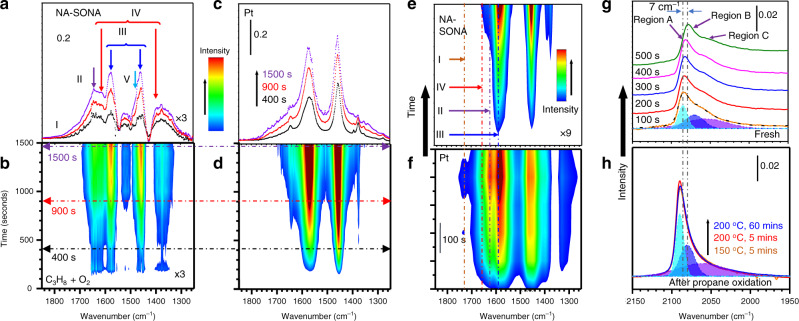
In situ DRIFTs data for (PtNiCo)^core^(PtNiOCoO)^surface^. Time-resolved DRIFTs spectra over PtNiCo–PtNiOCoO/Al_2_O_3_ (*n* = 42, *m* = 39, 1.0 wt%, ×3) and Pt/Al_2_O_3_ (1.0 wt%) at 250 °C under propane/oxygen (~5:1) within 1500 s. **a** The DRIFTs spectra and **b** the corresponding contour map for propane oxidation over PtNiCo–PtNiOCoO/A_2_O_3_. **c** The DRIFTs spectra and **d** the corresponding contour map for propane oxidation over Pt/A_2_O_3_. The contour map of DRIFTS spectra during propane oxidation over PtNiCo–PtNiOCoO/Al_2_O_3_ (*n* = 42, *m* = 39, 5.0 wt%, **e**, ×9) and Pt/Al_2_O_3_ (5.0 wt%, **f**) at 350 °C within 600 s; and the DRIFTs spectra monitoring CO desorption by purging N_2_ over CO-pretreated PtNiCo–PtNiOCoO/Al_2_O_3_ (*n* = 42, *m* = 39, **g**), and the spectra at 500 s N_2_ purging after CO pretreatment following propane oxidation (**h**): (orange) 150 °C for 5 min; (red) 200 °C for 5 min, and (blue) 200 °C for 60 min.

Given the significance of surface-oxygenated sites, we examined the formation of surface intermediate species during catalytic propane oxidation over PtNiCo–PtNiOCoO/Al_2_O_3_ (*n* = 42, *m* = 39, 1.0 wt%) and Pt/Al_2_O_3_ (1.0 wt%) at 250 °C (~20% propane conversion) by in situ DRIFTs (Fig. 4a–d). In comparison with the dominance of Type III species over Pt catalyst, Type II–V species were detected over PtNiCo–PtNiOCoO catalyst (Fig. 4a, b). The apparent distribution ratio of the different species in terms of the sum of the peak area integral (*A*), as determined by $\mathrm{DR} = A(\mathrm{LT}){\mathrm{/}}A(\mathrm{HT})$, where LT represents the lower-temperature active Type II, IV, and V species, whereas HT represents the higher-temperature Type I and III species (based on a previous study of reactivities of these species on Pt catalysts^24^), reveals DR ~ 6.3 for PtNiCo–PtNiOCoO and ~2.2 for Pt after 1500 s reaction (Supplementary Fig. 16a). This is indicative of propensity of formation of LT species on NA–SONA catalysts. The results were also substantiated by in situ DRIFTs data for the catalysts (5.0 wt%) under 350 °C (100% conversion) (Fig. 4e, f). In comparison with the presence of both LT and HT species over Pt (DR ~ 0.84), only HT species were detected over PtNiCo–PtNiOCoO as a result of complete removal of LT species (Fig. 4c, d and Supplementary Fig. 16b).

The surface sites were also probed by CO desorption on the catalyst at RT (Fig. 4g, h), revealing surface species in three frequency regions. The peaks at ~2085 (A), ~2070 (B), and ~2050 cm^−1^ (C) are characteristic of CO linearly adsorbed on oxygenated Pt^*δ*+^ sites, Pt^0^ terrace sites, and edge or kink sites, respectively. The red shift (~7 cm^−1^) after 500 s N_2_ purging for all peaks (Fig. 4g) was due to the reduction of dipole–dipole coupling^6^. After propane oxidation for 5 min at 150 °C, peaks in regions A, B, and C were blue-shifted by ~10, ~20, and ~30 cm^−1^, respectively. The increased CO adsorption capacity is evidenced by doubling of peak intensity, in contrast to the reduced adsorption capacity on Pt catalyst^11,24^. The CO adsorption characteristics showed little change after further propane oxidation at 200 °C for 1 h.

Given the surface oxygenation of Ni and Co, a partial charge transfer occurs on Pt sites upon introduction of propane, i.e., Pt^0^ to Pt^*δ*+^, and Pt^*δ*+^ to Pt^*n*+^ (1 > *n* > *δ*), through the surface-oxygenated Ni and Co species^30^. This assessment is supported by X-ray photoelectron spectroscopy (XPS) analysis of fresh and hydrothermally aged (800 °C) PtNiCo–PtNiOCoO/Al_2_O_3_ (*n* = 42, *m* = 39) (Supplementary Fig. 17). The relative surface composition and partial positive charge were shown to remain largely unchanged. This assessment is consistent with the finding that Ni and Co are distributed in the nearest neighbor of Pt atoms throughout the nanoparticles. During catalytic sweeping loops by varying O_2_/C_3_H_8_ ratios a hysteresis was revealed and maximized at 5:1 ratio (Supplementary Fig. 18), revealing a higher activity in the backward sweep than the forward one, which is opposite for Pt catalyst^24^. It confirms the higher stability of Pt-sites on the catalyst. The oxycarbon intermediates accumulation rate (OAR, s^−1^) for propane oxidation over PtNiCo–PtNiOCoO (Supplementary Fig. 19) exhibits an oscillatory behavior (±10^−2^ s^−1^), corresponding to a dynamic saturation of the surface intermediates.

In situ DRIFTs spectra for the PtNiCo–PtNiOCoO catalysts derived from other compositions, e.g., *n* = 25 and *m* = 56, and *n* = 82 and *m* = 1 (Supplementary Fig. 20a) were also analyzed, revealing subtle differences in the intermediates distribution and the oscillatory behavior of OAR (Supplementary Fig. 20b). Analysis of the tail gas composition (Supplementary Fig. 21) confirmed the better activity and stability for the PtNiCo–PtNiOCoO/Al_2_O_3_ (*n* = 42, *m* = 39) catalyst in comparison with that for Pt catalyst. For *n* = 25 and *m* = 56 (Supplementary Fig. 22), varying reaction temperature showed negligible impact on the distribution and breathing pattern of the surface intermediates. The characteristic of the time-dependent peak intensity (Supplementary Fig. 23) confirmed again the oscillatory characteristic of reactivity over NA–SONA catalysts. For reactant R (propane), intermediate I (LT and HT species), and product P (CO_2_), first-order reaction kinetics ($R\mathop { \to }\limits^{k1} I\mathop { \to }\limits^{k2} {\mathrm{P}}$) in terms of the apparent formation rate constants *k*_1_ and *k*_2_ (Supplementary Fig. 23) were analyzed. *k*_*2*_ is negligibly small. The formation for LT (*k*_1_ ~ 5.5 × 10^−4^ s^−1^) is faster than that of HT species (*k*_*1*_ ~ 4.7 × 10^−4^ s^−1^) over the ternary catalyst. This contrasts with the result with Pt catalyst (4.5 × 10^−4^ s^−1^ for LT and 9.0 × 10^−4^ s^−1^ for HT species).

### Comparing propane oxidation kinetics between NA–SONA and Pt

Taken together, the sharp differences in the structural and activity data for propane oxidation between PtNiCo–PtNiOCoO and Pt catalysts indicate that alloying Ni and Co with Pt likely paves a reaction pathway significantly different from Pt catalyst. This assessment is supported by the higher activity, higher deactivation resistance, different distribution of surface intermediate species, and a reaction-induced irregular oscillatory behavior for intermediates growth/removal in response to the atomic structure disordering and Pt–Pt bond distance expansion for the NA–SONA catalyst. The “regular–irregular oscillatory kinetics” captured by our in situ/operando characterizations has in fact provided for the first time the experimental evidence for the results from earlier Monte Carlo simulation of the irregular/regular oscillatory kinetics for CO oxidation following Mars–van Krevelen mechanism over nm-sized catalysts^31^. It was shown that the irregularity of oscillation would increase when the particle size is reduced to ~4 nm. The oscillatory kinetics in terms of period and magnitude display a tendency of transition from regular to irregular oscillations as the size shrinks, which results from size- and temperature-dependent random fluctuations of reactions on small particle surfaces. In our case, the particle size falls at the boundary of regular and irregular regions.

In addition, given the fact that aliphatic ester and acetone species detected on Pt were not detected over the NA–SONA catalysts, supporting an effective C–C cleavage, part of the experimental findings can also be understood based on the adsorption energies of propane species upon ^α^C–H or ^β^C–H bond cleavage (Supplementary Fig. 24a), calculated from density functional theory (DFT) calculation on cluster models. A minimum in adsorption energy is revealed for ^α^C–H and ^β^C–H bond cleavage when *n* ~ 6 in Pt_*n*_(NiCo)_(13−*n*)_ cluster. This indicates that with Ni and Co in the catalyst, ^β^C–H bond cleavage of propane is more favored over the Pt sites, facilitating the complete C–C cleavage and the formation/removal of LT species, which agrees with the observation from in situ DRIFTs measurement. The calculated adsorption energies for two exemplary intermediates, e.g., enolate (LT) and acetate (HT) (Supplementary Fig. 24b), show a clear difference (1.47 eV (on Pt) and 2.70 eV (on Ni), respectively), suggesting that enolate is weakly adsorbed on Pt site while acetate is strongly bonded to Ni or Co, depending on the composition. In comparison, the difference in adsorption energy for enolate (1.90 eV) and acetate (2.40 eV) on pure Pt is much smaller. The propensity for ^β^C–H bond cleavage of propane, weaker adsorption energy of LT species, and strong bonding of HT species on Ni or Co sites led to the Mars–van Krevelen pathway. The creation of the active Pt^*δ*+^ center originates from maneuvering the oxygenation degree of Ni or Co in the ternary site (Supplementary Fig. 24c). Indeed, the monitoring of the transients of reactant and product in propane stream over the pre-oxygenated ternary alloy catalysts revealed a clear decrease of propane and an increase of CO_2_, in sharp contrast to the largely silent feature for the Pt catalyst, demonstrating that the surface, subsurface or lattice oxygen is the leading active species in catalytic reaction (see Supplementary Fig. 11). This type of surface/subsurface sites enables active and stable catalytic cycles through lattice oxygen without coking. In contrast, the pure Pt surface site on Pt catalyst does not provide such catalytic synergy, and is thus prone to coke formation and deactivation.

In conclusion, the combined in situ/operando DRIFTs and HE-XRD/PDFs study has enabled us to unravel the significance of the NA–SONA structure of the ternary catalyst in the activity enhancement and deactivation resistance during catalytic propane oxidation. This structure features surface-oxygenated (PtNiOCoO)^surface^ species responsible for the oscillatory surface species and a long range structural disordered alloy core responsible for maintaining the structural integrity and stability. The coupling of a partial positively charged Pt with the oxyphilic Ni–O and Co–O species is shown to play an important role in the active surface for the cleavage of carbon–carbon bonds of the adsorbed propane and the effective removal of reaction intermediates on the surface. Further understanding of the nanostructure responsible for this active site synergy will be aided by theoretical modeling, as in previous work using DFT calculation to reveal the increase of adsorption energies and the decrease of reaction barriers for CO oxidation over Pt alloy catalysts^32^, and in the assessment of thermodynamics at the nanoscale^33^ in terms of the nanoscale phase properties that are known to differ from those of the bulk counterpart. In contrast to conventional Pt catalysts suffering from the propensity of PtO formation, coke formation, and aggregation-induced deactivation, the design of NA–SONA catalysts constitutes a promising pathway to overcoming these problems. The refinement of the surface oxygenation strategy may lead to a paradigm shift in the design of active and stable catalysts for catalytic oxidation reactions.

## Methods

### Chemicals

Platinum (II) acetylacetonate (Pt(acac)_2_, 97%) and nickel (II) acetylacetonate (Ni(acac)_2_, anhydrous, >95%) were purchased from Alfa Aesar. Cobalt (III) acetylacetonate (Co(acac)_3_, 99.95%) was purchased from Strem Chemicals, and 1,2-hexadecanediol (90%), benzyl ether (99%), oleylamine (70%), and oleic acid (99+%) were purchased from Sigma Aldrich. γ-phase aluminum oxide pellet (43832) was purchased from Alfa Aesar and kept under ball milling conditions for 48 h before further use. Vulcan carbon XC-72 was obtained from Cabot. Overall, 5.0 wt% or 1.0 wt% Pt/Al_2_O_3_ catalysts used in this work were also purchased from Alfa Aesar as the control catalysts. Gases of propane (1 vol% balanced by Argon), O_2_ (20 vol% balanced by N_2_), and H_2_ (99.99%) gases were purchased from Airgas. All chemicals were used as received.

### Catalysts preparation

The synthesis of PtNiCo nanoparticles involved the reduction and decomposition of three metal precursors, Pt^II^(acac)_2_, Ni^II^(acac)_2_, and Co^III^(acac)_2_, in controlled molar ratios in a benzyl ether solvent at elevated temperature modified according to previous work^26,27^. During the synthesis, oleylamine and oleic acid in control ratios were used as capping agents and 1,2-hexadecanediol as a reducing agent. Briefly for synthesis of Pt_45_Ni_33_Co_22_, 4.88 mmol of 1,2-hexadecanediol, 0.3 mmol of Co(acac)_3_, 0.3 mmol of Ni(acac)_2_, 0.3 mmol of Pt(acac)_2_, 1 mmol of oleylamine, 1 mmol of oleic acid, and 200 ml benzyl ether were added to a three-neck 500 ml flask under vigorous stirring and nitrogen gas atmosphere. The above solution was first heated to 100 °C under nitrogen purge and then heated to 270 °C, and refluxed for 40 min. The nanoparticle product was cleaned by ethanol and dispersed in hexane for further use.

The resulted nanoparticles were further supported on support materials (e.g., Al_2_O_3_, or carbon) before further use for catalytic reactions. For the preparation of Al_2_O_3_ or carbon supported nanoparticles, a typical procedure involved suspending 1–5 g Al_2_O_3_ or carbon in 10 ml hexane containing ~10–50 mg nanoparticles followed by stirring for ~12 h. The resulted powder was collected and dried under N_2_. The supported catalysts were further treated in a quartz tube furnace. The catalyst was calcined at 260 °C in 15 vol% O_2_ for 30 min noted as the (PtNiCo)^core^(PtNiOCoO)^surface^, simply PtNiCo–PtNiOCoO, catalysts. This synthesis-processing protocol resulted in all samples having virtually the same trimetallic composition to the nanoparticles. In this study, 0.25 wt%, 0.5 wt%, 1.0 wt%, and 5.0 wt% supported PtNiCo–PtNiOCoO catalysts were prepared and tested. Characterizations of the PtNiCo–PtNiOCoO nanoparticles with different compositions by HE-XRD indicated that these nanoparticles are the fcc-type structure characteristic of a solid solution^34,35^.

### Catalytic activity measurements

The catalytic activity of the catalysts for propane (1 vol% balanced by helium) + O_2_ (20 vol% balanced by N_2_) was measured using a customer-built testing station including a temperature-controlled quartz tubing reactor, gas flow/mixing/injection controllers, and an online gas chromatograph (Shimadzu GC 8A) equipped with 5A molecular sieve and Porapak Q packed columns and a thermal conductivity detector. The catalysts were loaded in a quartz microreactor tube (inner diameter: 4 mm) and wrapped by quartz wool in the middle of the tube (length of the catalyst bed: 6 mm). The feeding gas (1 vol% propane + 20 vol% O_2_ gases in 1:1 ratio) was injected continuously through the fixed catalyst bed in the quartz microreactor at a flow rate of 50 ml min^−1^. The residence time was about 0.09 s. Gas hourly space velocity used in this work is ~40,000 h^−1^. The catalytic activity for propane oxidation reaction was determined by analyzing the composition of the tail gas effusing from the quartz microreactor packed with catalyst fixed bed.

### Morphology and structure characterizations

To determine the morphology of the PtNiCo NPs and catalysts, TEM measurements were carried out on a JEM 2100F from JEOL operated at 120 kV. HAADF-STEM equipped with energy dispersive X-ray spectrometer was performed on a JEOL-ARM200F instrument with an acceleration voltage of 200 kV and a routine nominal resolution of 0.8 Å. The samples were prepared by dropping cast of hexane suspension of nanoparticles or supported nanoparticle catalysts onto a carbon-coated copper grid followed by solvent evaporation and plasma treatment at RT before measurement. ICP-MS spectrometer equipped with a simultaneous extended dynamic range detector was used to analyze the metal composition and metal loading, carried out on a PerkinElmer Elan 6000. Laboratory check standards were analyzed every eight samples, with instrument re-calibration if check standards were not within ±5% of the initial concentration. TGA was performed on a PerkinElmer Pyris 1-TGA for determining the nanoparticle concentration and metal loading for supported catalysts. A Physical Electronics Quantum 2000 scanning ESCA microprobe was used to conduct XPS experiments. An excitation source of focused monochromatic Al Kα X-ray (1486.7 eV), a 16-element multichannel detection system, and a spherical section analyzer were equipped on the instrument. A 100 µm diameter X-ray beam was rastered over a 0.2 mm by 1.4 mm rectangular spot on the sample. The binding energy was calibrated by C 1 s peak at 284.8 eV.

### Diffuse reflectance infrared Fourier transform spectroscopy

In situ DRIFTS measurements over Al_2_O_3_ supported catalyst under propane oxidation reaction conditions were performed under ambient pressure on a Bruker Vertex 70 FTIR spectrometer equipped with a liquid nitrogen cooled MCT detector and a Praying Mantis^TM^ Diffuse Reflectance Accessory (Harrick Scientific Products, Inc.). Briefly, the sample cup in Praying Mantis^TM^ Diffuse Reflectance Accessory was filled up with around 30 mg fine powders of Al_2_O_3_ supported catalysts. The sample cell was then heated up to reaction temperature (e.g., 250 °C) under helium and kept purging for at least 10 min before background collection followed by exposure to a mixture of propane, oxygen balanced by helium gas atmosphere at a constant total flow rate of 80 ml min^−1^ with C_3_H_8_ to O_2_ at stoichiometric ratio ~1:5. The purity of compressed oxygen/helium tank and 4.01% propane/helium tank were 99.999%. Each DRIFTs spectrum was collected with a nominal resolution of 2 cm^−1^ and 128 scans at every 100 s. Water compensation was conducted for all spectrum before further analysis. All samples were milled for ~30 min before use.

In situ CO adsorption and desorption measurements were also conducted over Al_2_O_3_ supported catalyst at RT. Briefly, pure helium gas was firstly introduced to cell chambers for 10 min before background collection followed by purging CO/helium at ~40 ml min^−1^ at which the CO adsorption spectra were recorded every 100 s at a sum of 128 scans for each spectrum until fully saturated. CO adsorption spectra were recorded by switching back to helium atmosphere.

### Combined DRIFTs and high-energy synchrotron X-ray diffraction coupled to pair distribution function analysis

A customer-modified FTIR instrument and Praying Mantis Diffuse Reflectance Accessory DRIFTs cell were used that enable simultaneous collections of DRIFT spectra and HE-XRD data, respectively, at Sector 11-ID-B of Argonne National Laboratory. Briefly, a Bruker Vertex 80 FTIR instrument was firstly mounted in perpendicular to X-ray beam, with a pre-aligned penetration hole at a dimension of 1 cm in diameter through the instrument to transmit the incident X-ray. A parallel slot was made in the sample cell for X-ray transmission, which was sealed by Kapton film before loading catalyst samples. The reaction process follows firstly heating up to reaction temperature (e.g., 250 °C) under helium at 40 °C min^−1^, then switched to a constant flow containing 5 ml min^−1^ 3 vol% C_3_H_8_ balanced by helium and 15 ml min^−1^ 5 vol% O_2_ balanced by helium after kept under helium for ~10 min at reaction temperature and lastly cooling back down to RT under propane/oxygen reaction atmosphere. During the reaction process, DRIFTs spectra were collected with a nominal resolution of 2 cm^−1^ and a sum of 128 scans every 100 s in continuous mode whereas synchrotron HE-XRD data were taken every 4–5 min using X-rays with a wavelength of 0.1378 Å (X-ray energy of ~95 keV), and the tail gas was analyzed by RGA residual gas analyzer at intervals of 7 seconds. Experimental XRD data were further corrected for experimental artifacts, reduced to the so-called structure factors, *S(q)*, and then Fourier transformed to atomic PDFs *G*(*r*) using a wave vector defined as *q* = 4πsin(*θ*)/*λ*, where *θ* is half of the scattering (Bragg) angle and *λ* is the wavelength of X-rays used. The simulated atomic PDFs were further derived based on Pt alloy fcc models. Note that, as derived, atomic PDFs *G*(*r*) are experimental quantities that oscillate around zero and show positive peaks at real space distances, *r*, where the local atomic density ρ(*r*) exceeds the average one ρ_o_. This behavior can be expressed by the equation *G*(*r*) = 4πrρ_o_[ρ(*r*)/ρ_o_ − 1], which is the formal definition of the PDF *G*(*r*). Ex situ HE-XRD experiments were also conducted over Alumina or carbon supported multimetallic catalyst samples in thin-wall glass capillaries with a diameter of 1.5 mm. The experimental XRD data were taken at RT under ambient atmosphere.

### X-ray absorption fine spectroscopy

XAFS spectra of Pt L3-edge (11,564 eV), Co K-edge (7709 eV), and Ni K-edge (8333 eV) were collected at beamline 9-BM-B of APS, Argonne National Laboratory. Powder as-prepared catalysts samples were pressed into pellets before measurement. Fitting of EXAFS spectra were performed using Artemis. The fitting was limited to 2.0–16.0 Å^−1^ for Pt L3-edge spectra and 2.0–12.5 Å^−1^ for Ni and Co K-edge spectra, using a Hanning window with dk = 1.0 Å^−1^. The first coordination shell fitting was performed in the region of 1.0 < *R* < 3.2 Å to both the real and imaginary parts of *χ*(*R*).

### DFT calculation

Ab initio calculations were carried out by DFT calculation as implemented in Dmol^3^ package of Materials Studio (Accelrys Inc.). The Perdew–Burke–Ernzerhof function and a double-numerical basis set with polarization functions were applied in all DFT calculations. In this work, a 13-atom cluster model was performed with complete geometry optimizations in all atomic configurations in which all atoms were fully relaxed. The interactions between atomic configurations of the cluster models and adsorbed molecules including CH_3_CH_2_CH_2_, CH_3_CHCH_3_, CH_2_CH–O, and CH_3_COO were investigated. The adsorption energy of each species on the cluster models was determined by ∆*E*_ads_ = *E*_adsorb_ − *E*_alloy_, where *E*_adsorb_ and *E*_alloy_ are the total energy for alloy system adsorbed with individual species (e.g., CH_3_CH_2_CH_2_, CH_3_CHCH_3_, CH_2_CH–O, and CH_3_COO) and pure alloy system, respectively, as a measure of the adsorption strength.

## Supplementary information

Supplementary Information

Peer Review

## Data Availability

All the relevant data are available from the authors upon request.
