# Supplementary material for: Surface oxygenation of multicomponent nanoparticles toward active and stable oxidation catalysts
Source: Nat Commun. 2020 Aug 21;11:4201. doi: 10.1038/s41467-020-18017-3 (PMC7443134; doi:10.1038/s41467-020-18017-3)
Supplement: Supplementary file 1 — Supplementary Information [file 41467_2020_18017_MOESM1_ESM.pdf]

Supporting information

Surface oxygenation of multicomponent nanoparticles toward active and stable oxidation catalysts

Shan et al

Supporting information

Surface oxygenation of multicomponent nanoparticles toward active and stable oxidation catalysts

Shiyao Shan[1], Jing Li[1], Yazan Maswadeh[2], Casey O'Brien[3,†], Haval Kareem[1,3], Dat T. Tran[3], Ivan C. Lee[3], Zhi-Peng Wu[1], Shan Wang[1], Shan Yan[1], Hannah Cronk[1], Derrick Mott[4], Lefu Yang[5], Jin Luo[1], Valeri Petkov[2,*], and Chuan-Jian Zhong[1,*]

[1] Department of Chemistry, State University of New York at Binghamton, Binghamton, NY 13902, USA

[2] Department of Physics, Central Michigan University, Mt. Pleasant, Michigan 48859, USA

[3] CCDC Army Research Laboratory, FCDD-RLS-CC, 2800 Powder Mill Road, Adelphi, MD 20783

[4] School of Materials Science, JAIST, Nomi, Ishikawa, 923-1211, Japan

[5] College of Chemistry and Chemical Engineering, Xiamen University, Xiamen 361005, China

†Present address: College of Engineering, University of Notre Dame, Notre Dame, IN 46556, USA

*Correspondence to: cjzhong@binghamton.edu, petkolvg@cmich.edu.

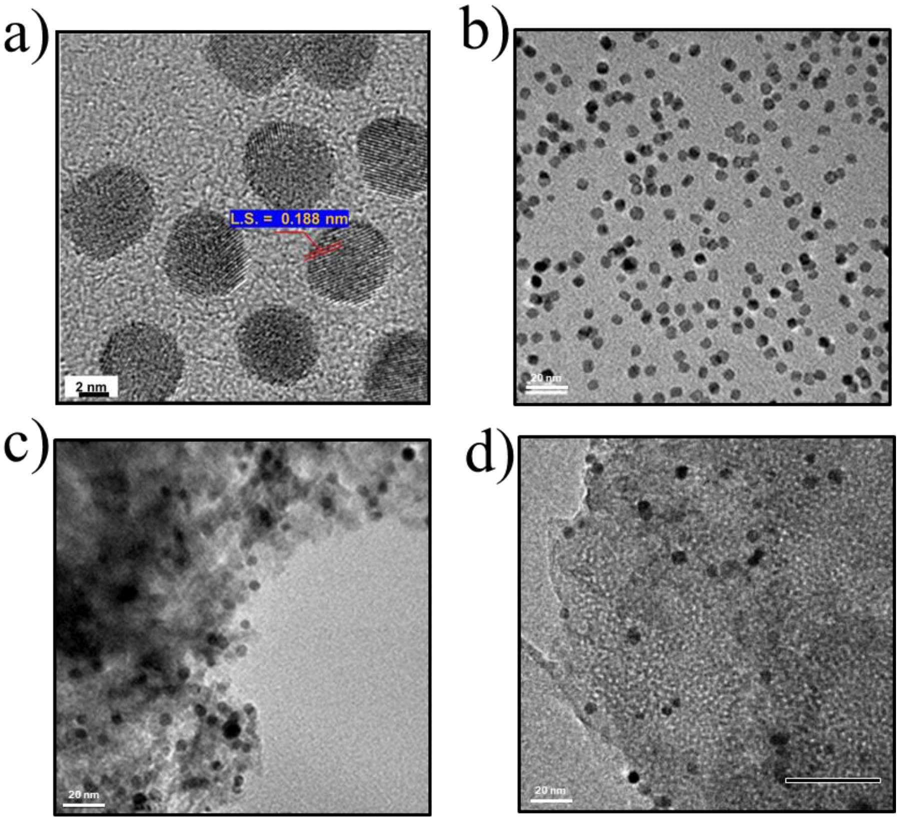

Supplementary Fig. 1. A representative set of HR-TEM (a) and TEM images (b): $Pt_nNi_mCo_{100-n-m}$ (n= 42, m = 39) nanoparticles and Al_2O_3 supported PtNiCo–PtNiOCoO catalysts (n= 42, m= 39): (c) 5.0 wt%; and (d) 1.0 wt%.

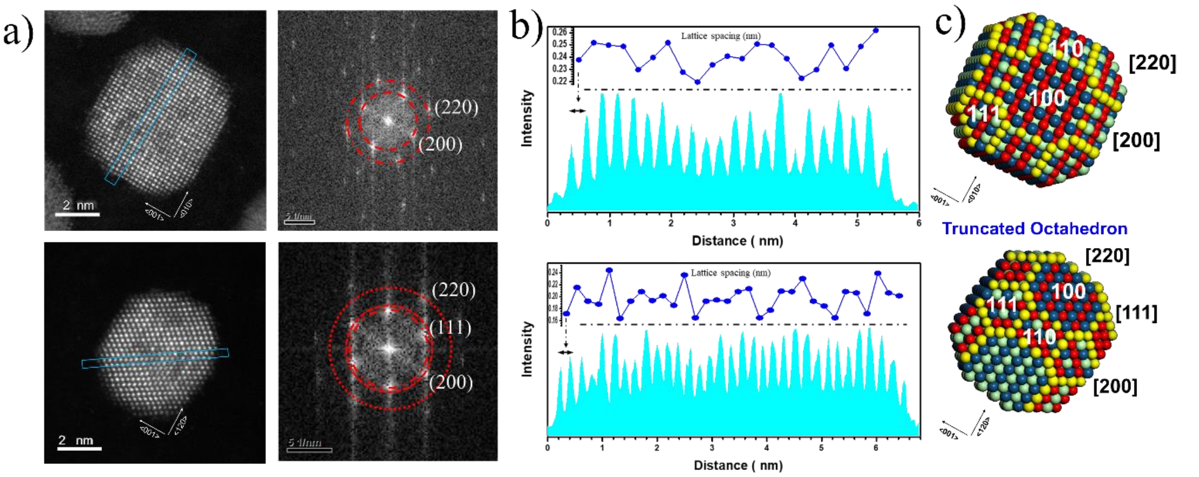

Supplementary Fig. 2. Morphologies of PtNiCo ternary alloy. (a) Aberration-corrected HAADF-STEM images and corresponding fast Fourier transform (FFT) patterns for as-synthesized $Pt_nNi_mCo_{100-n-m}$ (n=42, m =39) (<100> (top) and <110> (bottom) zone axes indicated); (b) Analysis of lattice patterns along the indicated area of STEM images (a); (c) illustration of truncated octahedron nanoparticles (Pt in blue, Ni in green and Co in red, with yellow highlighting the boundary between the facets).

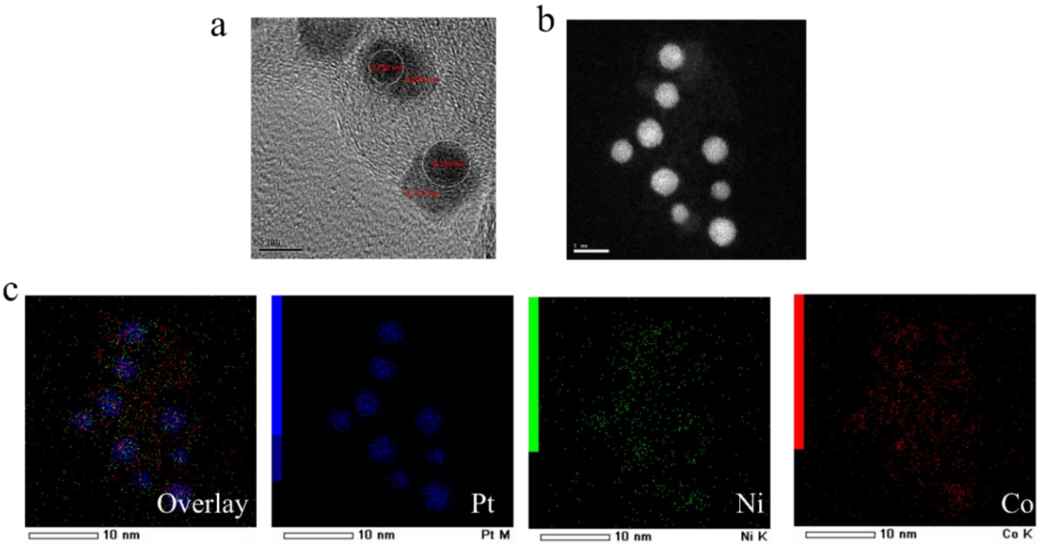

Supplementary Fig. 3. Morphologies of carbon black supported (PtNiCo)^{core}(PtNiOCoO)^{surface} catalyst: a representative set of HR-TEM (a) and HAADF-STEM images (b) and the corresponding EDX elemental mapping in b: Pt (blue), Ni (green) and Co (red) (c).

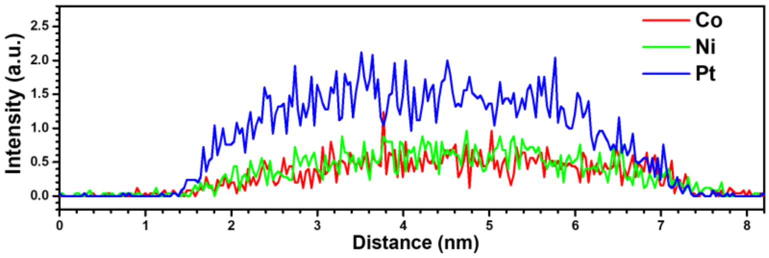

Supplementary Fig. 4. EELS line profiles for the three different metal components corresponding to the EDX mapping data shown in Fig. 1**b**.

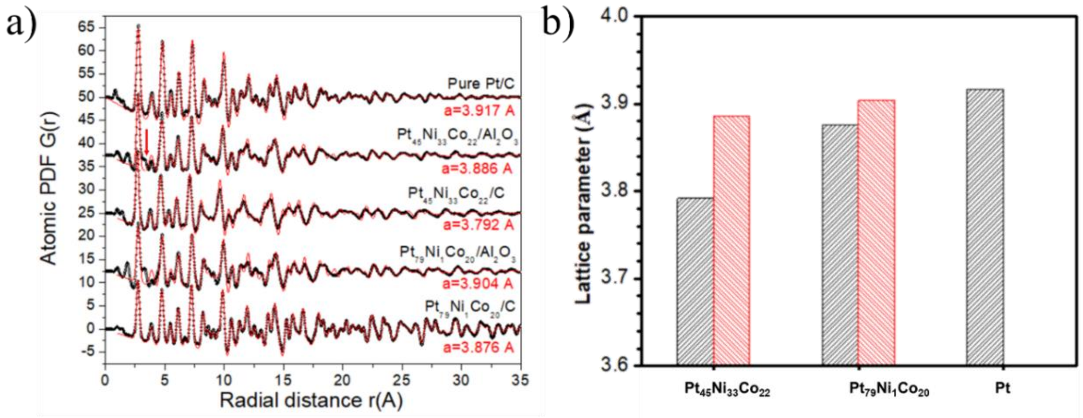

Supplementary Fig. 5. HE-XRD/PDFs: (a) Experimental PDFs (black dots) and model simulated PDFs (red curve) derived from HE-XRD data over Al_2O_3 and carbon black supported PtNiCo–PtNiOCoO catalysts in comparison with Pt/C catalysts. Red arrow: "satellites" peaks on both sides of the first PDF peak which is attributed to presence of limited surface oxidation; and (b) The plot of lattice parameter as a function of trimetallic composition for carbon and alumina supported catalyst extracted from HE-XRD/PDFs analysis in (a). Note that the normalized strain $\Delta\alpha/\alpha$ reference to lattice parameter of pure Pt catalysts were determined by $\Delta\alpha/\alpha = (\alpha_{Pt/Al2O3\ or\ C} - \alpha_{PtNiCo/Al2O3\ or\ C})/\ \alpha_{Pt/Al2O3\ or\ C}$, where $\alpha_{PtNiCo/Al2O3}$ and $\alpha_{PtNiCo/C}$ are the lattice parameters for Al_2O_3 supported and carbon black supported PtNiCo–PtNiOCoO catalysts, respectively. The standard deviation is ±0.0076 Å.

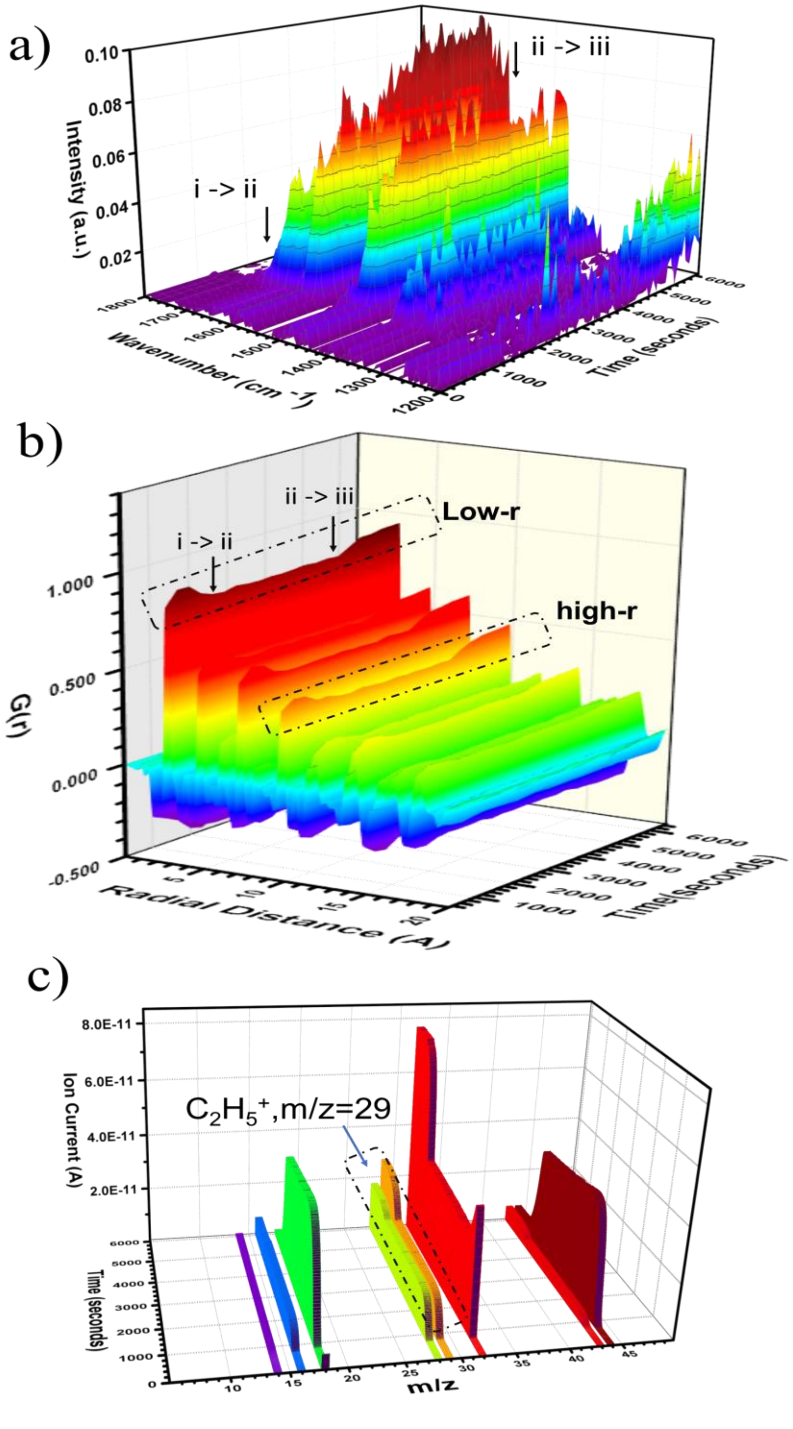

Supplementary Fig. 6. 3D plots of the full spectra corresponding to the in-situ/operando DRIFTS, HE-XRD/PDF and MS data shown in Fig. 3. (a) In-situ DRIFTS spectra (catalyst loading 5.0 wt%) operated at 250 ºC for 1 hrs followed by cooling back to RT for 30 mins; Major intermediates identified: (I) 1732 cm^{-1} (linear adsorption of aliphatic ester (R-C(=O)O-)); (II) 1650 - 1645 cm^{-1} (bridge adsorption of bicarbonate ($HOCOO^-$) or stretching mode of adsorbed H_2O); (III) 1590 - 1576 cm^{-1} (υ_{as}(R-COO^-)), 1458 cm^{-1} (υ_s(R-COO^-)) and 1376 cm^{-1} (δ_{CH}) linked to bridge adsorption of acetate (CH_3COO^-) or formate ($HCOO^-$); (IV) 1626 - 1615 cm^{-1} (υ_{as}), 1398 cm^{-1} (υ_s) and 1337 cm^{-1} (δ_{CH}) (atop adsorption of enolate (CH_2=CHO^-)); and (V) 1470 cm^{-1} (υ_{as} (CH_3-O^-)), linear adsorption of methoxy species(CH_3-O^-)) (b) In-situ PDF patterns simultaneously collected at interval of 3 mins; (c) 3D contour map of corresponding tail gas analysis by online mass spectrometer collected every 12 seconds..

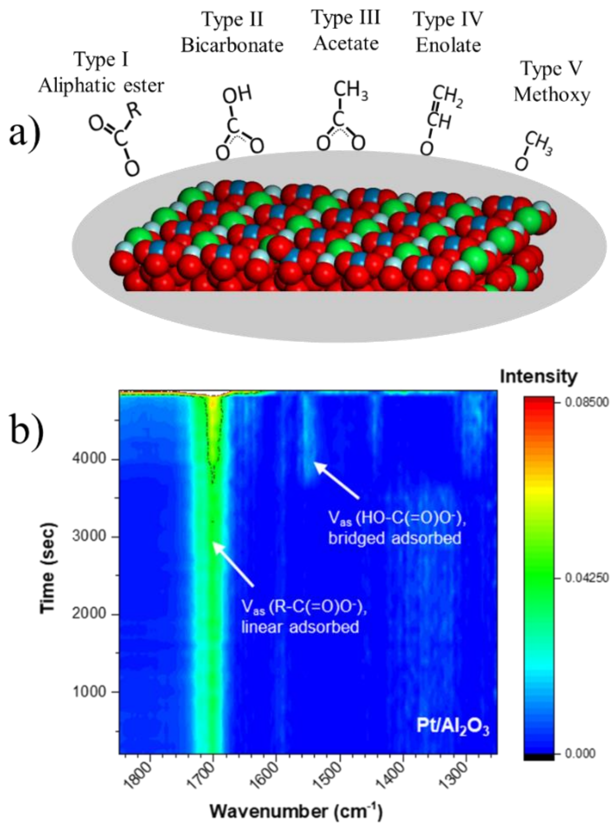

Supplementary Fig. 7. (a) Illustration of the major types of surface intermediate species of propane oxidation over a model surface of PtNiCo–PtNiOCoO/Al_2O_3: Types I – V; (b) 2D Contour map of time-resolved, in-situ DRIFTS spectrum over 5.0 wt% Pt/Al_2O_3 operated at 250 °C for 1 hr at propane-to-oxygen ratio equal to 1:5 followed by cooling back to room temperature for 30 mins.

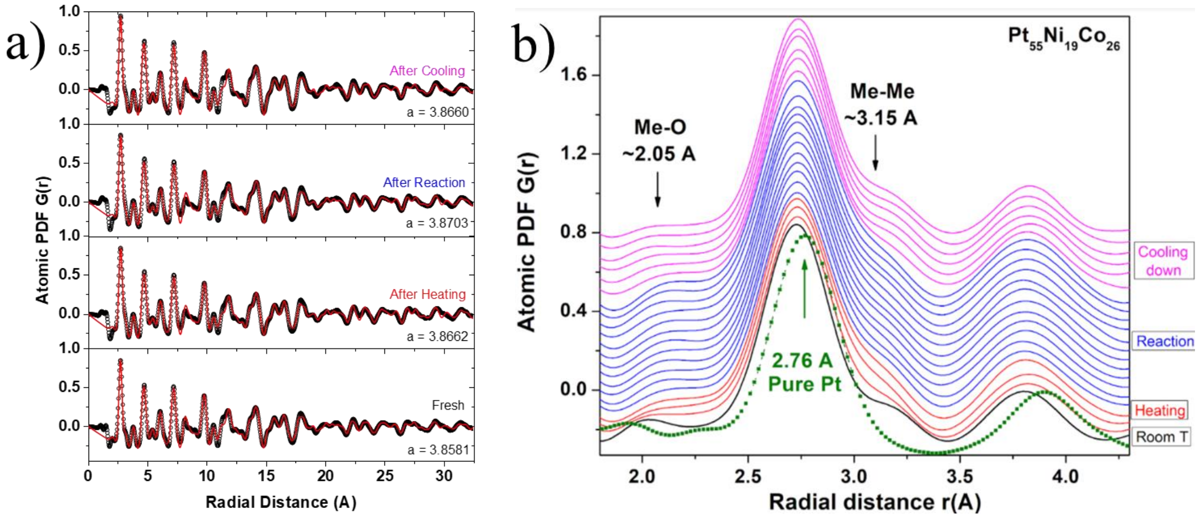

Supplementary Fig. 8. (a) A representative set of experimental PDFs (black dots) and modeled PDFs (red line) for the PtNiCo–PtNiOCoO catalyst corresponding to the different stages of the reaction ((i), (ii) and (iii)); (b) Zoom-in view of the atomic PDFs patterns in the region of Pt-Pt first bonding distance for PtNiCo–PtNiOCoO catalysts.

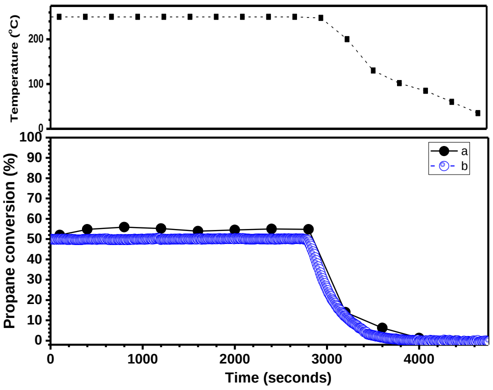

Supplementary Fig. 9. Time stream of propane oxidation over PtNiCo-PtNiOCoO catalyst. Plots of both in-house (by GC, a) and in-situ catalytic activity (by MS, b)) as a function of time over 5 wt% PtNiCo-PtNiOCoO catalysts during propane oxidation at 250 °C for 1 hr followed by cooling back to room temperature.

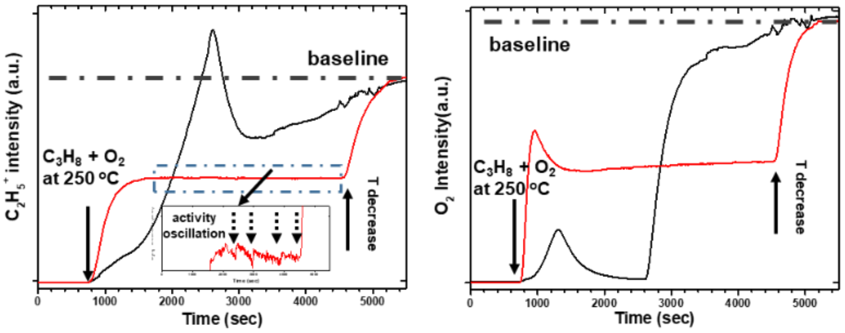

(A) (B)

Supplementary Fig. 10. Plots of ion current intensity of $C_2H_5^+$ (A) and O_2^+ (B) fragments as a function of time by examination of the tail gas using online mass spectrometer over Al_2O_3 supported PtNiCo–PtNiOCoO (red) and Pt (black) catalysts.

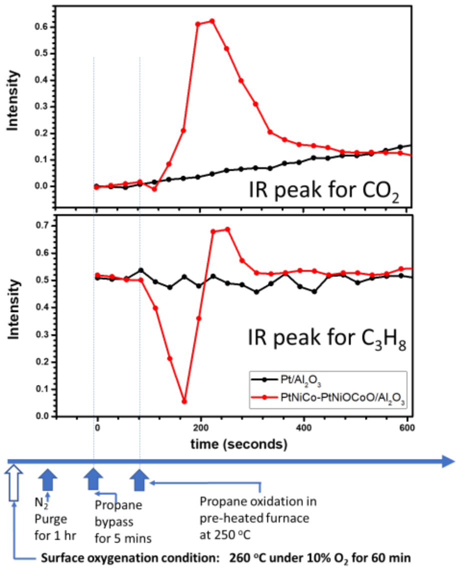

Supplementary Fig. 11. Plots of catalytic activity (in FTIR peak intensities of reactant and product) vs. reaction time upon purging propane feeding gas only (without O_2) through the oxygenated catalysts: PtNiCo catalyst and Pt catalyst. The reaction was monitored under N_2 protected atmosphere following purging propane feeding gas (0.2 vol%) through N_2 protected catalysts atmosphere under 250 °C for 20 mins.

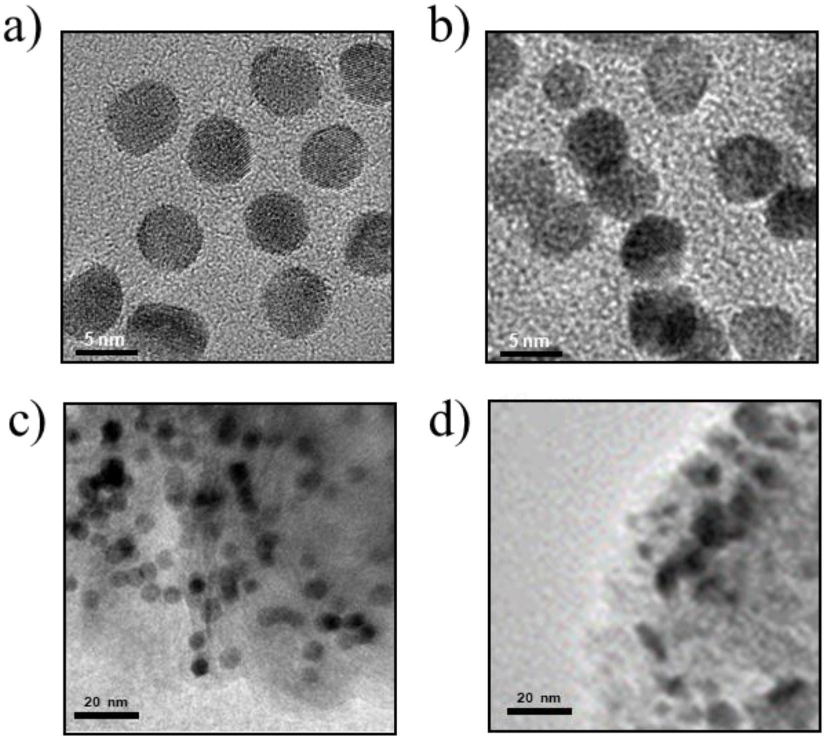

Supplementary Fig. 12. HR-TEM images for as-synthesized $Pt_nNi_mCo_{100-n-m}$ (n= 22, m = 29) (a, particle size 6.1 (± 0.5) nm) and (n= 82, m = 1) (b, particle size ~5.5 (± 1.0) nm) nanoparticles, and the TEM images for the corresponding PtNiCo–PtNiOCoO/Al_2O_3 catalysts (5.0 wt %) (n= 22, m= 29) (c, particle size ~6.7 (± 0.4) nm) and (n= 82, m= 1) (d, particle size 7.8 (± 1.0) nm).

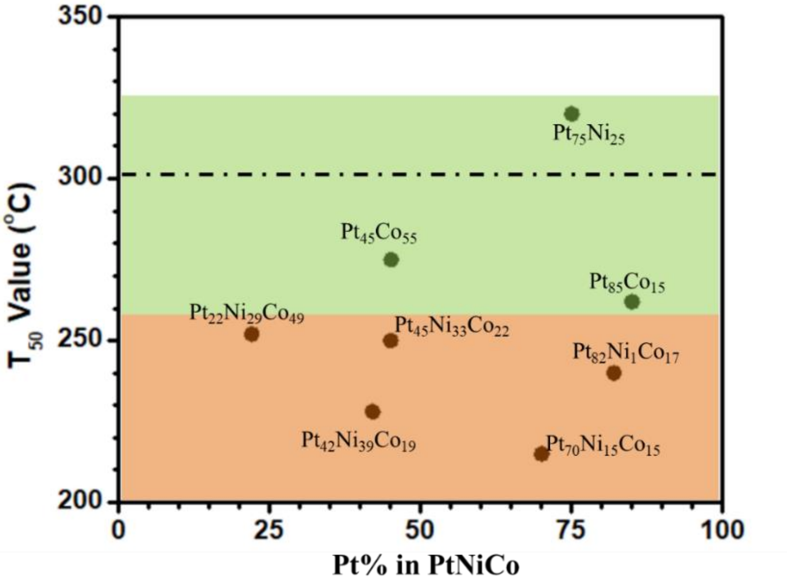

Supplementary Fig. 13. Plots of T_{50} value as a function of Pt% in the catalysts in comparison with PtNi and PtCo, e.g., PtNiCo–PtNiOCoO/Al_2O_3 catalysts (5.0 wt%) (atomic ratio of Pt= 22 (a, black), 42 (b, red), 45 (c, blue), 70 (d, dark green), and 82 (e, pink), where, atomic ratio of Co = 29 (a, black), 39 (b, red), 33 (c, blue), 15 (d, dark green), and 1 (e, pink)), in comparison with $Pt_{85}Co_{15}$ (f, green, T_{50} ~ 262 °C), $Pt_{75}Ni_{25}$ (g, brown, T_{50} ~319 °C) and $Pt_{45}Co_{55}$ (h, gray, T_{50} ~275 °C); Note that dash line indicate the catalytic activity of commercial Pt (5.0 wt%) catalysts, where orange region shows the T_{50} values for the PtNiCo–PtNiOCoO catalysts are all lower than those in the green region for the binary counterparts and commercial Pt catalysts. The standard deviation is ±0.5 °C

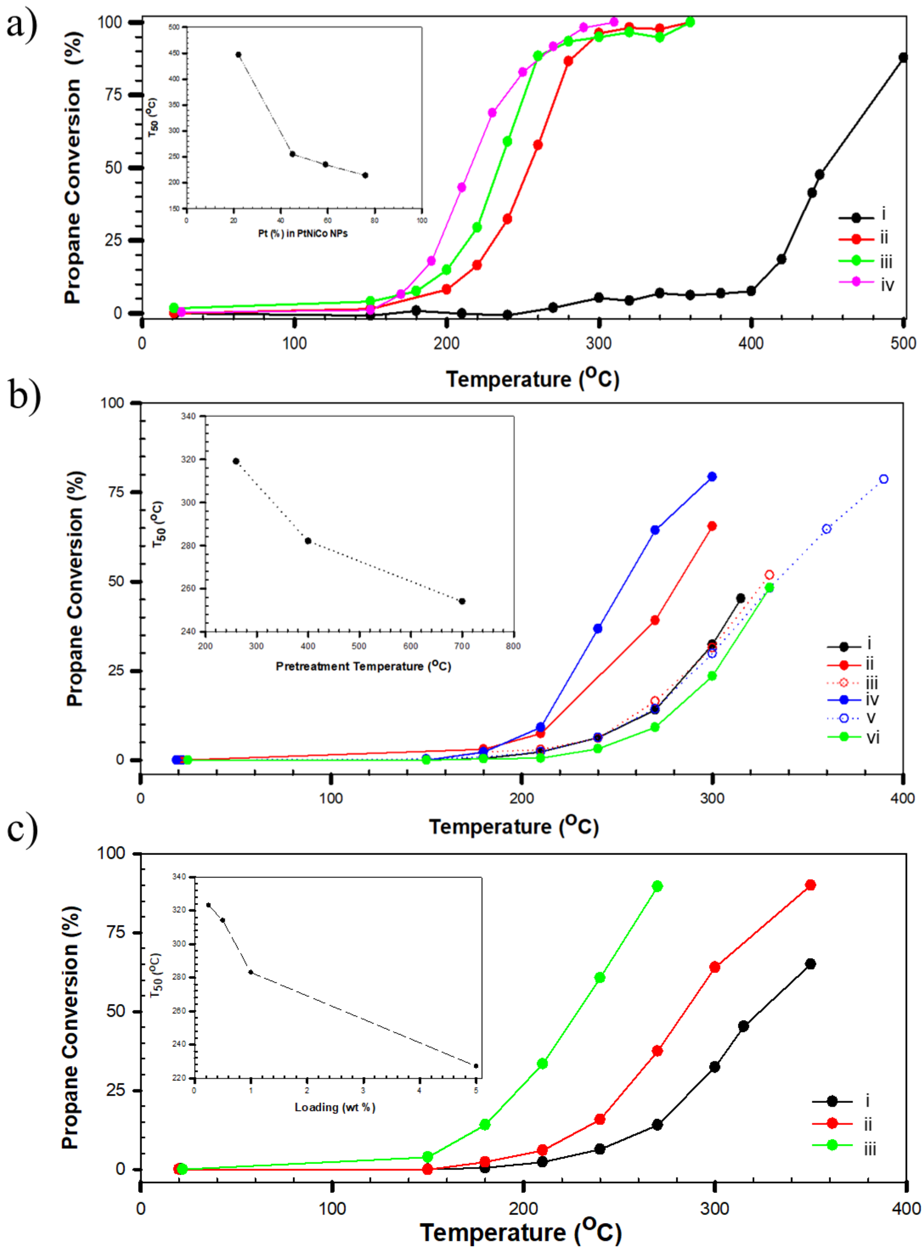

Supplementary Fig. 14. (a) Propane conversion (in %) over PtNiCo–PtNiOCoO/C catalysts (15 wt%): n= 22, and m= 74 (black, i); n= 45, and m= 33 (red, ii); n= 59, and m = 17 (green, iii); and n = 82, m = 1 (pink, iv), inset: Plot of T_{50} value as a function of Pt% in the catalysts; (b) Propane conversion (in %) over PtNiCo–PtNiOCoO/Al_2O_3 (n=42, m = 39, 0.25 wt% total metal loading) catalysts treated under O_2 at 260 ℃ (i, black, T_{50} ~ 303 °C), 400 °C (ii, red solid, T_{50} ~284 °C); ambient atmosphere exposure for 30 days after 400 °C treatment(iii, red dash, T_{50} ~310 °C), 750 °C (iv, blue, T_{50} ~254 °C), ambient atmosphere exposure for 30 days after 750 °C treatment (v, blue dash, T_{50} ~317 °C), and H_2 treatment only at 400 °C (vi, green, T_{50} ~321 °C) inset: Plot of T_{50} values as a function of oxygen thermal treatment temperature; (c) Propane conversion (in %) over PtNiCo–PtNiOCoO/Al_2O_3 (n=42, m = 39): (i, 0.25% wt, black); (ii, 1.0 wt% wt, red), and (iii, 5.0 wt%, green), inset: T_{50} values as a function of total metal loading in wt%. The standard deviation is ±0.3%

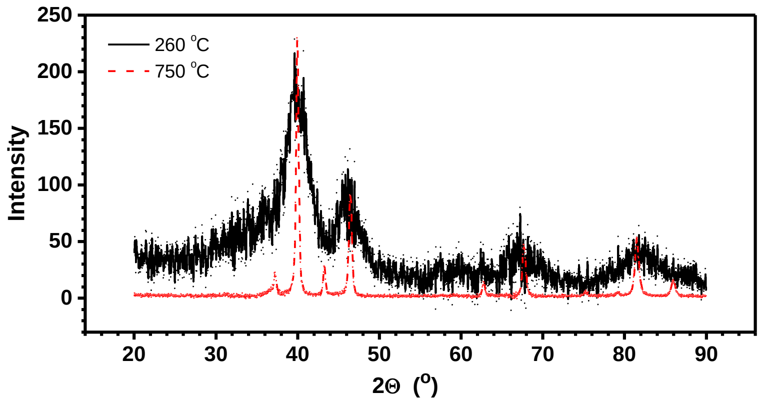

Supplementary Fig. 15. Powder XRD pattern of PtNiCo–PtNiOCoO/Al_2O_3 catalyst (10.0 wt% metal loading) after treatment under air at 260 °C for 1 hr (black) and under air at 750 °C (corrected for background Al_2O_3). Note that in order to obtain enough XRD intensity, a much higher metal loading, 10.0 wt%, was used instead of the regular low metal loading (<1%) used for the catalytic reaction, and in this case a significant aggregation occurred after 750 °C treatment under air. Nevertheless, the basic alloy characteristics largely remained, showing small peaks associated with oxygenated Ni and Co species.

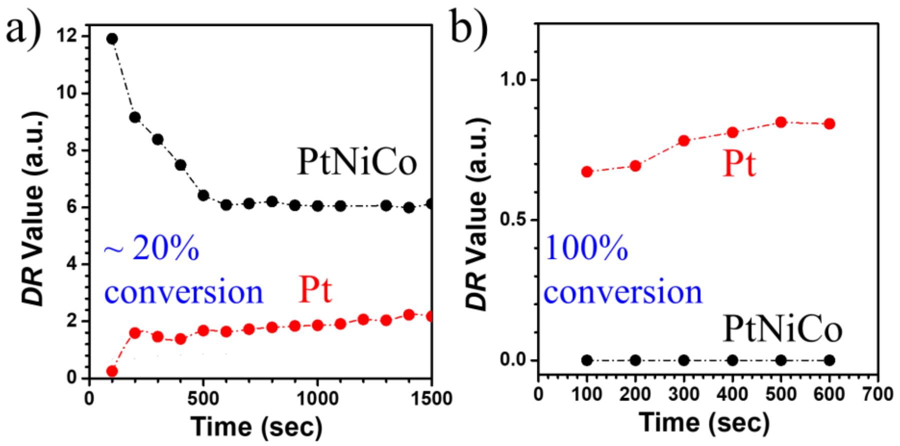

Supplementary Fig. 16. (a) Plots of *DR* values vs. time, which were determined by the ratio of the sum of peak areas extracted from the data in Figs. 4**a** – 4**b** associated with enolate, methoxy and bicarbonate (A_{LT}) to the sum of peak areas associated with acetate and alphabetic ester (A_{HT}) from deconvoluted DRIFTs spectra, for propane oxidation over Pt/Al_2O_3 (red) and PtNiCo–PtNiOCoO/Al_2O_3 (n=42, m = 39) (black) catalysts (1.0 wt%) at 250 °C. (b) Plots of *DR* values vs. time for propane oxidation over 5 wt% catalysts at 350 °C extracted from the data in Fig. 4**c**.

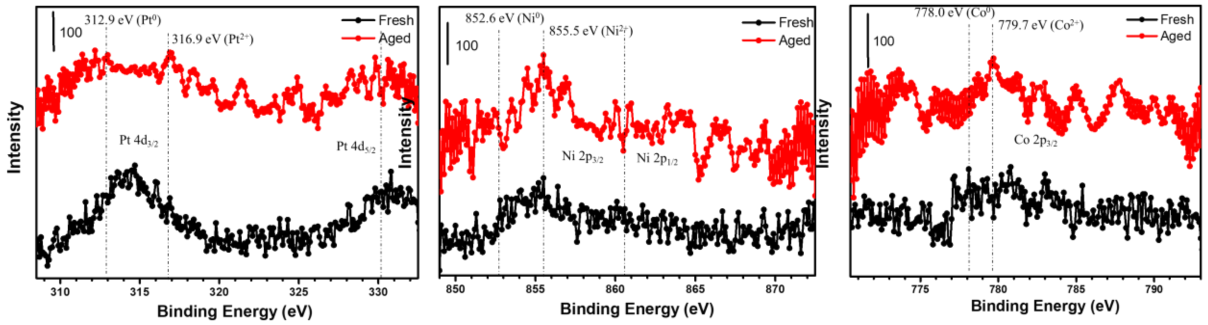

Supplementary Fig. 17. XPS spectra for fresh (black curve) and hydrothermally aged (red curve, treated at 800 °C under 10% CO_2 + 10% H_2O + N_2 for 16 hrs) PtNiCo–PtNiOCoO/Al_2O_3 (n=42, m = 39, 5.0 wt%) catalysts: (a) Pt 4d; (b) Ni 2p; and (c) Co 2p. Note that all spectra were calibrated by C 1s peak at 284.8 eV. Quantitative fitting of the spectra yielded little change in the relative surface composition change (from $Pt_{42}Ni_{42}Co_{16}$ for fresh catalyst to $Pt_{43}Ni_{36}Co_{21}$ for the aged one).

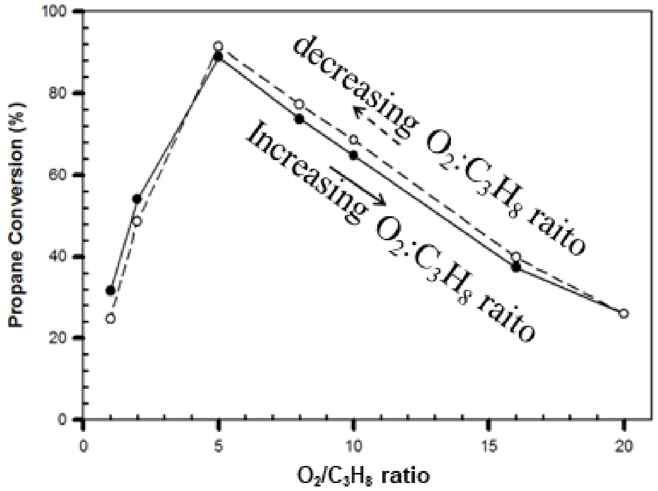

Supplementary Fig. 18. Propane conversion over PtNiCo–PtNiOCoO/Al_2O_3 (n=42, m = 39, 1.0 wt%) as a function of O_2 to C_3H_8 ratio in a loop forwarding from 0.25 to 20 and back to 0.25 at 300 °C: (solid line): increasing O_2/C_3H_8 ratio, (dash dots): decreasing O_2/C_3H_8 ratio.

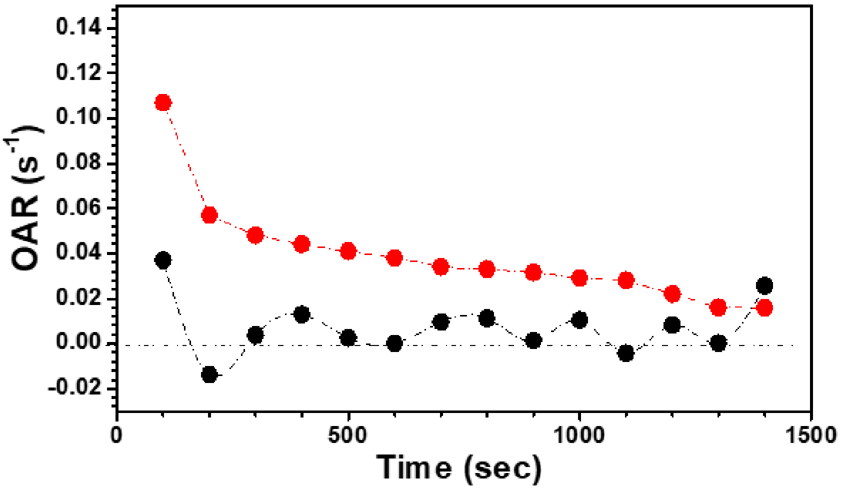

Supplementary Fig. 19. The apparent total oxy-carbon surface species accumulation rate (OAR, turnover frequency, s^{-1}) extracted from integral DRIFTs peak areas over PtNiCo–PtNiOCoO/Al_2O_3 (n=42, m = 39, 1% wt) catalyst in comparison with commercial Pt/Al_2O_3 catalyst in Fig. 2**a** and **b**. The data were calculated upon the subtraction of integral peak areas of successive DRIFTs spectra as a function of reaction time in the range of 1200 - 1800 cm^{-1} based on the equation: $OAR = \frac{A*S*M(alloy)}{\Delta t*m*D*L}$, where A represent the integral DRIFTs peak area in the range of 1200 – 1800 cm^{-1}, S is area to rate coefficient (~ 1.506 e^{-7}) report in previous literature[1], M(alloy) is the molecular weight of the alloy, Δt represents the time interval (100 seconds), m is the total mass of the catalysts (~ 30 mg), D is the catalysts dispersion (~ 12 - 22% dependent on particle size of catalysts with different compositions) and L is the metal loading (~ 1 wt%). Note that the negative value of OAR indicates the surface species did not accumulate on the surface.

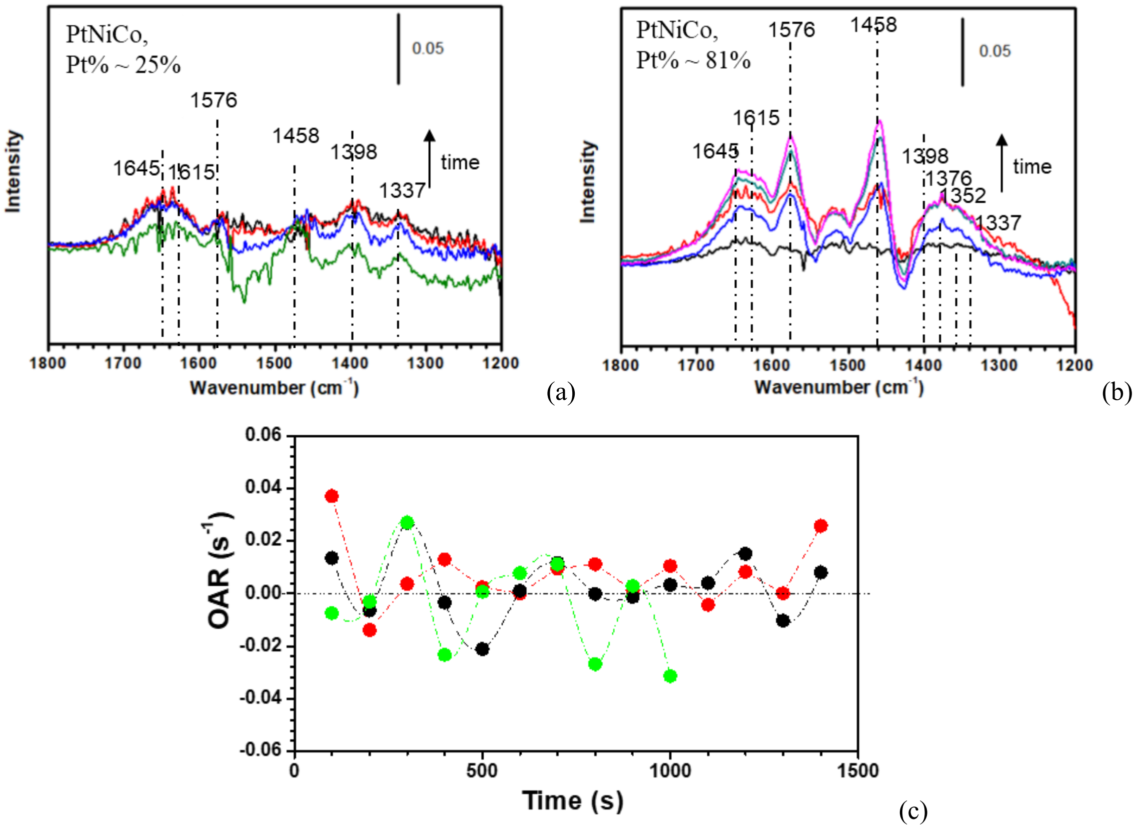

Supplementary Fig. 20. DRIFTS spectra for propane oxidation over 1 wt% PtNiCo–PtNiOCoO/Al_2O_3 (n=25, m = 56, a) and (n=81, m = 1, b) operated at 250 °C for 1 hrs under propane/oxygen reaction atmosphere at different times: 100 s (black), 500 s (red), 1000 s (blue), 1500 s (green) and 2000 s (purple); (c) The total surface oxy-carbon accumulation rate (turnover frequency, s^{-1}) extracted from peak areas of DRIFTs spectra at range of 1200 cm^{-1} to 1800 cm^{-1} in (a) and (b) for propane oxidation over PtNiCo catalysts, e.g., 1 wt% PtNiCo–PtNiOCoO/Al_2O_3 (n=25, m = 56, green), (n= 42, m= 39, red) and (n=81, m = 1, black) at 250 °C under propane oxidation reaction.

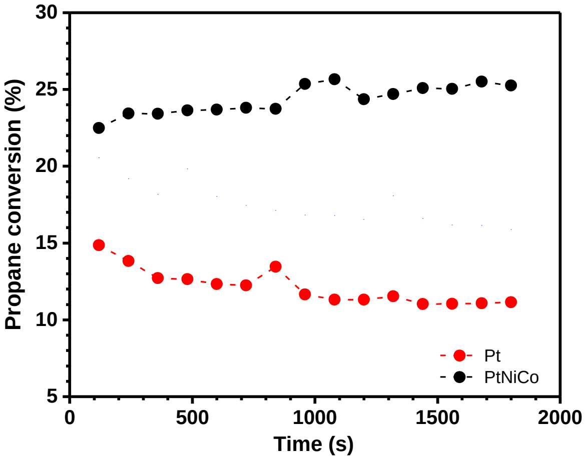

Supplementary Fig. 21. Propane conversion collected from an online gas chromatography analyzer over PtNiCo – PtNiOCoO/Al_2O_3 (n=42, m = 39, black) and commercial Pt/Al_2O_3 (red) during propane reaction process at 250 °C in the in-situ DRIFTs cell for 30 mins. The standard deviation is ±0.2%

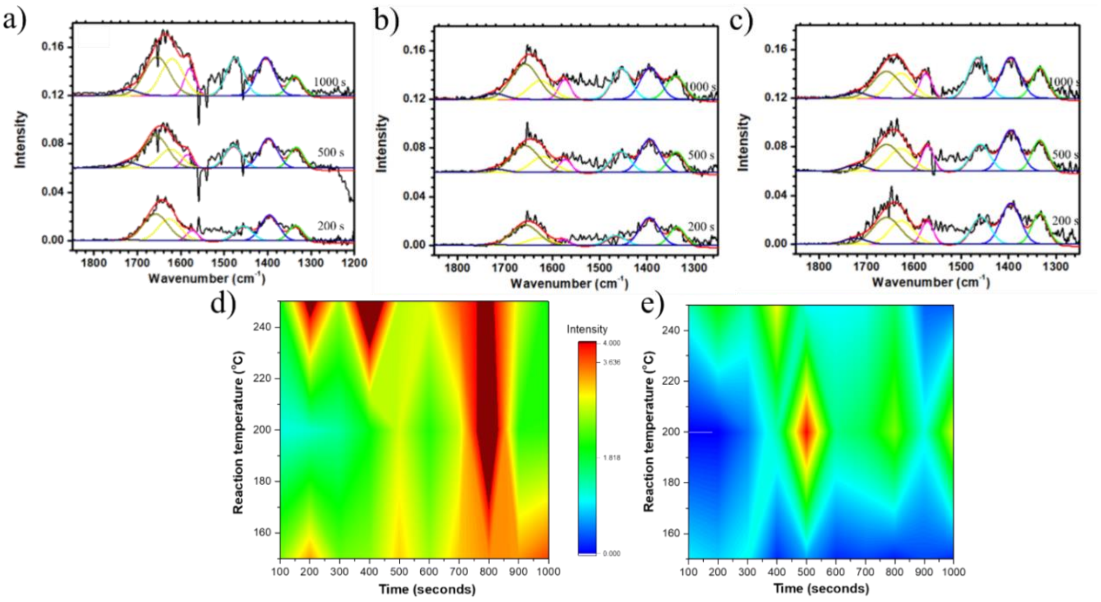

Supplementary Fig. 22. DRIFTS spectra of propane oxidation over PtNiCo–PtNiOCoO/Al_2O_3 catalyst (n=25, m = 56, 1.0 wt%) under different temperatures. Original (black dots) and deconvoluted (color lines) in-situ DRIFTs spectra collected at 150 (a), 200 (b) and 250 °C (c) after 200, 500 and 1000 s exposure to propane + O_2 atmosphere. (original spectrum data (black dots), the overall fitting curve (red) and individual peak fitting curves at 1650 (dark yellow), 1615 (yellow), 1589 (purple), 1459 (cyan), 1398 (blue), and 1337 cm^{-1} (green)); time revolved contour maps of in-situ DRIFTS peak area integral upon deconvoluted spectra as a function of reaction temperature for the peaks of 1650 (d, *LT*), and 1587 cm^{-1} (e, *HT*). The peak area integral is greater at 1650 cm^{-1} than that at 1587 cm^{-1} with a breathing pattern in every 300 – 400 seconds.

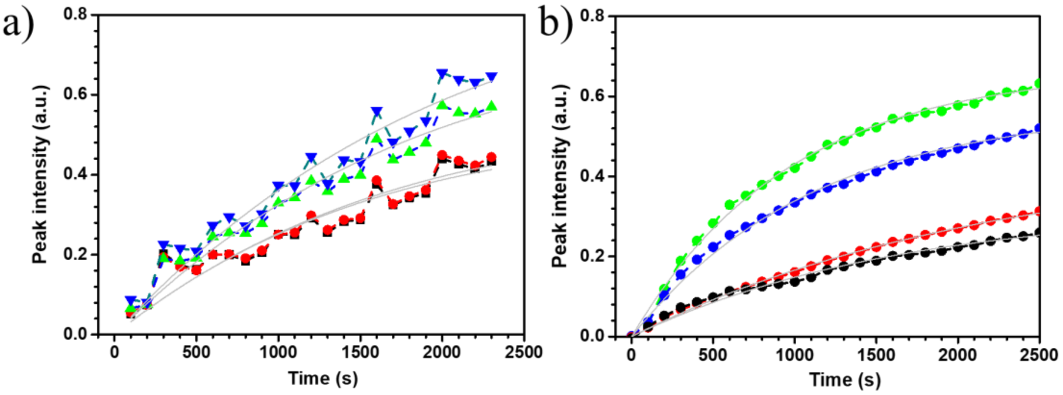

Supplementary Fig. 23. Plots of peak height intensity of the DRIFT peaks at 1650 (dark yellow), 1615 (red), 1589 (blue), 1576 (cyan green) cm^{-1} as a function of time for propane oxidation over PtNiCo–PtNiOCoO/Al_2O_3 (n=42, m = 39, 1.0 wt%, a) and Pt/Al_2O_3 (1.0 wt%, b). Dash color lines indicate the oscillatory pattern and gray solid lines indicate the first order kinetic fitting following the assumption that the intensity after 2500 s under propane oxidation is as close to steady state. For the reactions involving reactant R (propane), intermediate I (*LT* & *HT* species), and product P (CO_2), $R \xrightarrow{k1} I \xrightarrow{k2}$ P. Assuming that the reverse reaction is negligible, and all reaction steps follow the first-order kinetics, concentration I is expressed by $\frac{I}{C} = \left(\frac{k1}{k1-k2}\right)\left(e(-k2t) - e(-k1t)\right)$, where C represents the total species involved in the reactions, *k1* represents the apparent formation kinetic constant and *k2* represents the apparent dissociation kinetic constant. Given that *k2* is apparently negligible for surface intermediates, the above equation is simplified as $\frac{I}{C} = -e(-k1t)$. The apparent kinetic constants *k1* were determined by fitting the equation to the experimental data. Based on the fitting, the formation kinetic constant were determined to be ~5.5 x 10^{-4} s^{-1} for bicarbonate, 5.3 x 10^{-4} s^{-1} for enolate, 4.7 x10^{-4} $s^{-1}$for acetate and 4.3 x 10^{-4} s^{-1} for formate over PtNiCo catalysts, whereas the formation kinetic constant k1 for Pt catalysts were determined to be 4.5 x 10^{-4} s^{-1}, 6.1 x 10^{-4} s^{-1}, 9.0 x 10^{-4} s^{-1}, and ~1.0 x 10^{-3} s^{-1}, for bicarbonate, enolate, acetate and formate, respectively.

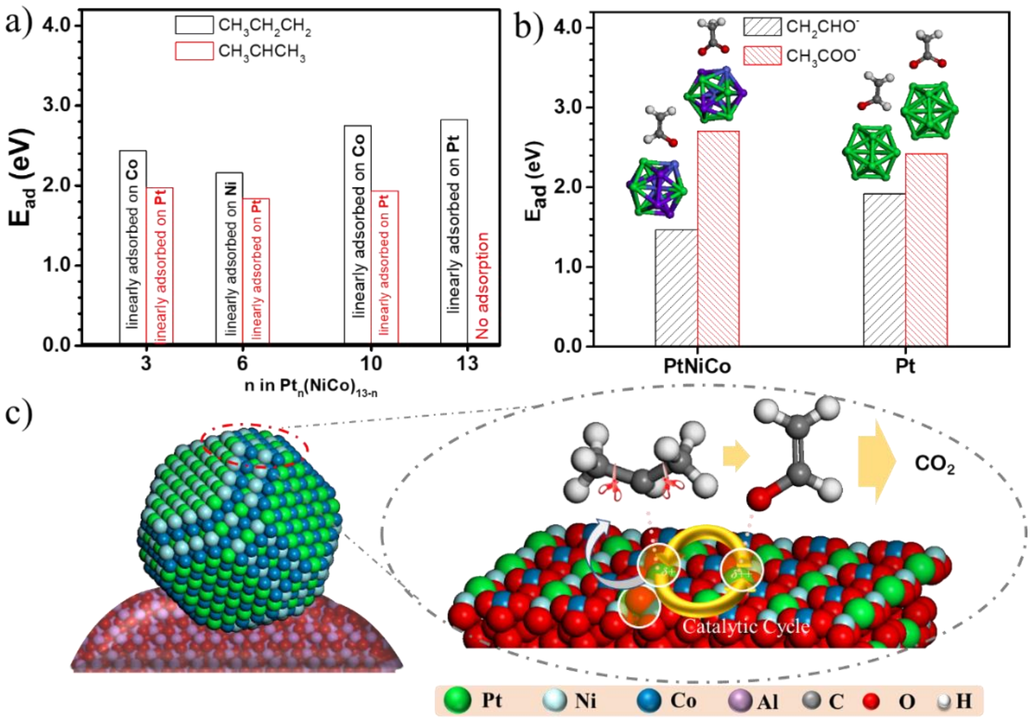

Supplementary Fig. 24. **Surface adsorption energies of intermediate species and reaction pathways**. (**a**) DFT calculated adsorption energy (E_{ad}) of -$CH_2CH_2CH_3$ ($^{\alpha}$C-H cleavage) and -$CH(CH_3)_2$ ($^{\beta}$C-H cleavage) over 13-atom PtNiCo cluster models with different composition of Pt; (**b**) Comparison of E_{ad} values of *LT* species (e.g., enolate, CH_2=CH-O^-) and *HT* species (e.g., acetate, CH_3-COO^-) over $Pt_6Ni_4Co_3$ and Pt_{13} cluster models; and (**c**) Illustrations of the catalytic cycle for propane oxidation over the surface-oxygenated ternary sites, where $Pt^{\delta+}$ atom is coupled to the oxophilicity of Ni or Co atoms as an active and stable site to promote a complete cleavage of carbon-carbon bonds of the adsorbed propane and an effective removal of reaction intermediates without coking.

Supplementary Table 1. CNs (M-O) (M = Pt, Ni, Co) extracted from EXASF analysis over PtCo, PtNi and $Pt_nNi_mCo_{100-n-m}$–PtNiOCoO/Al_2O_3 catalysts treated under H_2 first and then exposed to ambient before the analysis

Catalyst (n, m, 100-n-m)	CN (Pt-O)	CN (Ni-O)	CN (Co-O)
(45, 0, 55)	0.6±0.1	-	-
(64, 36, 0)	0.4±0.1	0.8±0.3	-
(50, 25, 25)	0.3±0.1	1.5±0.4	1.1±0.1

Supplementary Table 2. Lattice parameters (*a*) extracted from HE-XRD/PDF for $Pt_nNi_mCo_{100-n-m}$ –PtNiOCoO/Al_2O_3 and PtCo catalysts: as-prepared and treated at 260 °C under O_2.

Catalysts (n, m, 100-n-m)	*a* (As-prepared) (Å)	*a* (Treated at 260 °C under O_2) (Å)
(45, 0, 55)	3.878	3.896
(50, 25, 25)	3.813	3.864

Supplementary Table 3. Kinetic parameters for propane oxidation over $Pt_nNi_mCo_{100-n-m}$–PtNiOCoO/Al_2O_3 catalysts of different compositions in comparison with those derived from Pt, PtNi and PtCo catalysts.

Catalysts (n, m, 100-n-m)	d_{NP} (nm)	Metal loading	Reaction rate($\times 10^{-5}$ mol $g_{Pt}^{-1}.s^{-1}$)[a]	Ave. D (Dispersion, %)	TOF ($\times 10^{-2}$ s^{-1})[d]	Ave. D_o (Dispersion, (%)[e]	TOF_o (10^{-2} s^{-1})[f]	E_a (kJ mol^{-1})[g]
(22, 56, 22)	~6.1	5.0 %	8.35	15.4	10.5	14.1 (d=6.7 nm)	11.6	59.4
(42, 39, 19)	~4.9	0.25%	59.1[b]	19.2	60.0	15.2 (d=6.2 nm)	75.9	68.4
(42, 39, 19)	~4.9	0.5%	73.7[b]	19.2	74.8	15.2 (d=6.2 nm)	94.6	56.7
(42, 39, 19)	~4.9	1.0%	118.0[b]	19.2	119.7	15.2 (d=6.2 nm)	151.5	53.2
(42, 39, 19)	~4.9	5.0%	10.9	19.2	11.1	15.2 (d=6.2 nm)	14.0	42.2
(45, 33, 22)	~3.9	5.0%	6.46	24.1	5.2	/	/	50.0
(70, 15, 15)	~7.8	5.0%	9.91	12.1	16.0	12.1 (d=7.8 nm)	16.0	35.9
(82, 1, 17)	~5.5	5.0%	6.64	17.1	7.6	12.1 (d=7.8 nm)	10.7	29.9
(75, 25, 0)	~ 7.7	5.0%	4.04	12.2	6.4	/	/	73.3
(85, 0, 15)	~ 6.7	5.0%	4.48	14.1	6.2	/	/	71.8
(100, 0, 0)	~ 7.0	1.0%	31.9[b]	13.5	46.2	13.5 (d=7.0 nm)	46.2	40.0

Notes: [a] Calculation of reaction rate was based on moles of propane reacted at the flow rate and was normalized against the total mass of Pt in the catalyst. The standard deviation is ±0.06 x 10^{-5} mol g_{pt}^{-1}`s^{-1}. All data are extracted at 240 °C except notified [b] The data are extracted at 300 °C. The Pt specific dispersion on the surface of one NP referring to surface Pt over total available Pt species[2] was calculated based on a cone-shaped volume using $D=(6V_m/(a_m d_{NP}))\times 100\%$, where V_m is the volume of atom Pt (~ 0.0152 nm^3), a_m is Pt surface area (~ 0.096 nm^2), and d_{NP} is the particle size of alloyed nanoparticles determined by TEM. [d] Calculation of TOF was based on the surface Pt using TOF=R/(MW(Pt in the oxygenated alloy)×D), where R represent Pt specific reaction rate. [e] Calculation of dispersion (D_o) based on TEM-measured diameter (in parenthesis) for the oxygenated NPs. [f] Calculation of TOF_o based on D_o. [g] Data derived from temperature range at 200 – 300 °C from linear fitting of Arrhenius plot of ln(R) vs. 1/T (K) with R^2 value > 0.97, a slight deviation from linear relationship was observed.

Supplementary References:

1. C. P. O'Brien, G. R. Jenness, H. Dong, D. G. Vlachos, I. C. Lee, Deactivation of Pt/Al2O3 during propane oxidation at low temperatures: Kinetic regimes and platinum oxide formation. *J. Catal*. **337**, 122-132 (2016).
2. S. Y. Shan *et al.*, Oxophilicity and Structural Integrity in Maneuvering Surface Oxygenated Species on Nanoalloys for CO Oxidation. *ACS Catal.* **3**, 3075-3085 (2013).
