# Supplementary material for: Surface oxygenation of multicomponent nanoparticles toward active and stable oxidation catalysts
Source: Nat Commun. 2020 Aug 21;11:4201. doi: 10.1038/s41467-020-18017-3 (PMC7443134; doi:10.1038/s41467-020-18017-3)
Supplement: Supplementary file 2 — Peer Review [file 41467_2020_18017_MOESM2_ESM.pdf]

Reviewer #1 (Remarks to the Author):

It is a well-conducted piece of work reporting the dynamic valent state changes of Co and Ni in trimetallic PtCoNi NPs, and their consequences on oxidation reactions. The PtCoNi catalyst is indeed more active than Pt catalyst alone loaded on the same support. The characterizations are detailed and the origins of the increased activity is properly identified. I would recommend acceptance without revision should the paper is submitted to a specialized catalysis journal such as ACS Catal., the leading one in the area.

Considering Nat. Commun. has a much broader audience, I have to see whether the paper has broad enough implications. Although the authors claim their catalyst represents a different design approach, and "may represent a paradigm shift in the design of self-supported catalysts", I am not able to be convinced. The report herein is a rebranding of what has already been known before.

First of all, it is common knowledge that when PtM (M = 3d metal elements) is exposed to oxidative conditions, M on the surface will be oxidized, which then results in reorganization of the particle structure and electron distribution. The same applies to tertiary alloy particles such as PtCoNi studied in this paper. I do not see why this can be claimed as a design strategy for oxidation catalysts.

Secondly, the method does not have tuning flexibility, since the oxidation state and structure of the PtNiCo NPs depend on the thermodynamics. That is, we can not control the NP to stay in the best possible electronic/geometric state for a certain reaction. Rather, that structure is determined by the reaction condition (one reaction condition, one structure).

Another issue is the lack of control samples. It is a bit strange to see that the authors did not prepare and compare the structure and activity of PtNi and PtCo NPs.

Reviewer #2 (Remarks to the Author):

The authors present a very thorough study of propane total oxidation by ternary alloy nanoparticles. The breath of approaches applied to the problem and the number of samples studied is impressive. The authors conclude that the ternary alloy produces a more active catalyst by creating an active surface with mixed Ni and Co oxides decorated by Pt, as well as conclusions about the mechanism and instabilities in the kinetics. The conclusions are largely supported by the data, though some of the evidence in the main body of the paper is obscured by figures that are at best challenging to read. I have no doubts about the significance and novelty of the work, but considering its breath and the compromises in readability that the authors made to compress the paper to fit the page limits of a communication, I am not convinced this is the best venue for this paper. It would be much easier to follow and digest as a detailed long paper with all of the relevant figures in a single article, rather than needing to toggle to a supplement with 25 figures to understand what is going on. Below is a list of specific comments and questions the authors should address before publication.

1)The Introduction co-mingles partial and total oxidation but the authors do not make it clear that this paper is focused on total oxidation and the arguments they make about metal and oxide supports are relevant to total oxidation. In contrast, partial oxidation catalysts are most often oxides of earlier transition metals, e.g. V and Mo. The suggestion is that the authors clearly state their focus on total oxidation.

2) The ternary alloy phase diagram of Co-Ni-Pt is never mentioned but is extremely important. What is the miscibility range? Are there any ordered intermetallic phases? The description of the solid structure by a single fcc lattice constant suggests a solid solution, is this known to be the case over

the composition range of interest?

3) Line 95 – "further confirms the presence of the surface sites with Ni and Co atoms in the nearest neighbor of Pt atoms." It's not obvious how the figure tells us this, particularly since TEM sees through the entire particle. Since it is an important point the authors build on later on, it is important to explain how they came to this conclusion.

4) Figure 1 and the discussion surrounding it on page 3 – the surface layer in Fig 1a is not obvious and in the accompanying Fig. S3a the labels on the image were illegible on my copy. The discussion of strain, I don't understand the significance of the strain with respect to Pt, should be with respect to the bulk alloy with the composition of interest, and definitely need to indicate whether that strain is compressive or tensile.

5) Also on page 3, concerning the EXAFS analysis, in the fcc structure how do you get >12 nearest neighbors (Ni-Ni = 13.2)? I don't follow how the numbers lead to the suggestion of an ordered-layered structure. An ordered layered structure would not be consistent with a solid solution with an fcc structure. Review of information about what is known about the bulk ternary alloy would be helpful here.

6) Catalyst aging – the aging conditions are very extreme compared to their operating conditions, did the authors see any changes in activity with time on stream?

7) Figure 3 is so compressed that it is almost impossible to understand, it was only by looking in detail at the supplement that I was able to tell what was going on. The axis on the 3D plots are impossible to see, the color 3D IR spectra do not really show peaks that are easy to see when compared to the text, two of the three insets in the mass spec trace are impossible to see. The point about oscillations in the surface species does not come through at all, it is only in Figs. S19 and 21 that the reader can be convinced that this in fact occurs.

8) Mass spec data – m/z = 44 corresponds to the parent ions of both CO2 and propane. The authors should try to correct the m/z = 44 trace for the propane contribution by analyzing the cracking pattern, e.g. the 44/29 ratio when only propane is leaked into the mass spectrometer.

9) Page 7 Line 228 – "introducing lattice vacancy" where does this conclusion come from?

10) Page 9 Line 309 – "regular-irregular oscillatory kinetics" This term is used in several places, I don't know what it means.

11) Also on page 9, first time experimental evidence of oscillatory kinetics. As a blanket statement this certainly can't be true. Are they referring specifically to the Monte Carlo calculation referenced? This statement needs to be clarified.

Reviewer #3 (Remarks to the Author):

As the authors mentioned, oxidation reactions, total or partial oxidations, are widely used in the chemical industry and environmental governance, and the activation of gaseous oxygen is very important for these applications. The multi-component synergy of catalysts is one of the most important research topics in heterogeneous catalysis, and the interfacial interaction between different components is one of the key factors in determining the performance of catalysts. The present work reported an interesting phenomenon that the surface oxygenation of a multicomponent alloy of Pt with oxyphilic metals such as Ni and Co under catalytic reaction condition can generate multifunctional surface-active sites with substrate-active Pt center and surface oxygen-activating NiO/CoO sites. By

using in-situ/operando time-resolved characterizations, the authors successfully show reasonable explanation and make fruitful discussion. Some insights in this phenomenon is expected to generate wide interest in this area. I recommend it to publish after a minor revision. Here are some specific comments for consideration during the revision.

1.The authors called the surface oxygenation of Pt-alloyed multicomponent nanoparticles an Oxidation Catalysts. I cannot agree with it. In general, an oxidation catalyst means a non-metal oxidation catalyst. In the present work, the Pt-alloy is only partially oxidized.

2.Since the active sites are very complicated, the calculation of TOF is questionable. Can the oxidation degree be added as a correction factor? The authors used changes of TOF over different samples to support their concussion that that the catalytic activity originates from the ternary compositions, which needs further discussion and more evidences.

Is it possible that the surface oxidation of metal catalyst is common phenomenon? In the present work, what is the role of the alloy during self-oxidation. What happened for a single metal catalyst during the catalytic process?

3.The authors highlighted that the self-activated catalysts are stable oxidation catalysts. However, no related data is given to show what will happen if the used catalysts are pretreated in reduction atmosphere for reuse.

4.The discussions on reaction mechanism is a little confusing, especially on the Langmuir–Hinshelwood or Mars–van Krevelen pathway over different sites.

Point-by-point responses and revisions made in the manuscript (Manuscript ID: NCOMMS-20-17136)

REVIEWER COMMENTS

Reviewer #1:
It is a well-conducted piece of work reporting the dynamic valent state changes of Co and Ni in trimetallic PtCoNi NPs, and their consequences on oxidation reactions. The PtCoNi catalyst is indeed more active than Pt catalyst alone loaded on the same support. The characterizations are detailed and the origins of the increased activity is properly identified. I would recommend acceptance without revision should the paper is submitted to a specialized catalysis journal such as ACS Catal., the leading one in the area. Considering Nat. Commun. has a much broader audience, I have to see whether the paper has broad enough implications. Although the authors claim their catalyst represents a different design approach, and "may represent a paradigm shift in the design of self-supported catalysts", I am not able to be convinced. The report herein is a rebranding of what has already been known before.

Response: We thank the Reviewer's enthusiasm on the findings in our work. As for the broad implications, we have drawn a sharp contrast of our oxidation catalyst design to traditional oxidation catalyst design in the manuscript. Our design is the surface oxygenation of multimetallic alloy nanoparticles where the catalytic reaction occurs at the oxygenated active sites, in contrast to traditional metal/metal oxide support catalysts where the catalytic reaction occurs on metal/support perimeter zone. It is the oxygenated active site that we describe as self-supported catalyst because it does not require the traditional support. This has not been known before. For a better clarity, we further emphasized this contrast in the revised manuscript (p. 2).

- *First of all, it is common knowledge that when PtM (M = 3d metal elements) is exposed to oxidative conditions, M on the surface will be oxidized, which then results in reorganization of the particle structure and electron distribution. The same applies to tertiary alloy particles such as PtCoNi studied in this paper. I do not see why this can be claimed as a design strategy for oxidation catalysts.*

Response: Thanks for the comment. While it is a common knowledge that 3d metal element on the surface is oxidized when it is exposed to oxidative conditions, it is not well known how the degree of oxidation depends on the alloying composition and phase structures. Some 3d M may be less prone or even resistive to oxidation, or form a passivation layer or islands under oxidative conditions, depending on the chemical nature of the alloying. It is a new design strategy for oxidation catalysts because it directly creates the active sites from a pre-alloyed composition with much intimate atomic synergy, rather than the metal oxide supported catalysts where the active sizes are created at the metal/support perimeter zone. In this regard, relevant examples include CO oxidation over PdCo alloy catalysts where CoO_x on Pd serves active sites under the oxidative conditions (Ref. 14), and primary alcohol oxidation over metal/oxide such as FeO/Pt(111) where oxygen terminated interface aids O-H and C-H dissociations (New Ref 10, Nat. Comm., 8, 14039, (2017)). However, for our surface oxygenated PtM1M2, the alloy structure integrity remains while there is partial surface oxygenation of M1 and M2 for hydrocarbon oxidation. The oxygenated surface was evidenced by XASF and in-situ HE-XRD characterizations as described in our manuscript. With surface oxygenated alloying structure, both activity and stability in catalytic oxidation were achieved, as shown in Fig. 2 and the revised Supplementary Fig. 14B, all of which are not known in the literature. This concept is also in part theoretically supported by DFT calculation of CO oxidation over Pt-alloy catalysts, showing the increase of adsorption energies and the decrease of reaction barriers (New Ref 32, Catal. Tod. 165, 89, (2011)). For better clarity on our claim of the design strategy, we incorporated these comments into the revised manuscript (p.2. and p.10).

- *Secondly, the method does not have tuning flexibility, since the oxidation state and structure of the PtNiCo NPs depend on the thermodynamics. That is, we can not control the NP to stay in the best possible electronic/geometric state for a certain reaction. Rather, that structure is determined by the reaction condition (one reaction condition, one structure).*

Response: While we understand the Reviewer's point, we would like to emphasize that this method's tuning flexibility is actually much higher than the traditional method. First, as the reviewer pointed out, the structure indeed depends on the reaction condition. The controllability and tunability of the surface oxygenated structure is evidenced by the in-situ HE-XRD results where the lattice parameter is shown to be adjusted by the reaction temperature (See Fig. 3). Also, as shown in Supplementary Fig. 14B, the catalytic activity is shown to be greatly enhanced by increasing oxidative treatment temperature. The fact that the exposure to ambient atmosphere for an extended long period of time or reductive atmosphere leads to the initial activity indicates the surface oxygenated alloy structure is not only tunable but also stable under the catalytic oxidation condition. The alloy structure is shown to be mostly maintained even at treatment temperatures as high as 750 ^{o}C (See added Supplementary Fig. 15). Note that a metal loading as high as 10 wt% was used in order to obtain enough powder XRD signal. This high loading led to a significant segregation, which would not have occurred at the ultralow loading ($\leq$1%). Secondly, the tuning flexibility is also evidenced by the fact both the catalytic activity and the alloy structure can be tuned by the ternary composition (see Supplementary Fig. 13.). This tunability is reflected by the different degrees of surface oxygenation, e.g., different M-O coordination numbers, for PtNiCo of different oxidation degree (see Fig. 1). This level of structural tunability in surface oxygenation would not be possible in the case of the traditional supported catalysts. In the revision, we further highlighted this aspect with clearer articulation (p.8).

- *Another issue is the lack of control samples. It is a bit strange to see that the authors did not prepare and compare the structure and activity of PtNi and PtCo NPs.*

Response: While we appreciate the Reviewer's comment, we must point out that we have in fact included the results for PtNi and PtCo catalysts in Supplementary Fig. 13.and Supplementary Table 3 for comparisons, which indeed demonstrated clearly the better activity for the ternary PtNiCo catalysts. As shown by the results from XAS data comparison for PtNi and PtCo catalysts, less degrees of surface oxygenation of both Ni or Co species were found for PtNi and PtCo catalysts. In contrast, PtNiCo could maintain a higher degree of surface oxygenation even after reduction treatment (See added Supplementary Table 1). Such a high degree of surface oxygenation for PtNiCo catalysts was further confirmed in terms of the degree of the compressive strain by comparing fresh vs. surface oxygenated catalysts. While the exact lattice parameter depends on the composition and the treatment conditions, representative examples showed a change from 3.813 Å to 3.864 Å for PtNiCo catalyst, and 3.878 Å to 3.896 Å for PtCo catalyst after the surface oxygenation treatment (see added Supplementary Table 2). The degree of lattice expansion of the ternary catalyst is clearly greater (0.051 nm) than that of the binary catalyst (0.018 nm), reflecting the oxygen insertion into the lattice of the surface layer. For a better clarity on the control samples, we have some of the discussion into the revised version (p.3).

Reviewer #2:

The authors present a very thorough study of propane total oxidation by ternary alloy nanoparticles. The breath of approaches applied to the problem and the number of samples studied is impressive. The authors conclude that the ternary alloy produces a more active catalyst by creating an active surface with mixed Ni and Co oxides decorated by Pt, as well as conclusions about the mechanism and instabilities in the kinetics. The conclusions are largely supported by the data, though some of the evidence in the main body of the paper is obscured by figures that are at best challenging to read. I have no doubts about the significance and novelty of the work, but considering its breath and the compromises in readability that the authors made to compress the paper to fit the page limits of a communication, I am not convinced this is the best venue for this paper. It would be much easier to follow and digest as a detailed long paper with all of the relevant figures in a single article, rather than needing to toggle to a supplement with 25 figures to understand what is going on. Below is a list of specific comments and questions the authors should address before publication.

Response: We appreciate the Reviewer's recognition of the significance and novelty of our work, and the comments on the need of improvement of the figures. We have tried to improve the clarity of descriptions on the figures and combined a few figures for better understanding.

1) The Introduction co-mingles partial and total oxidation but the authors do not make it clear that this paper is focused on total oxidation and the arguments they make about metal and oxide supports are relevant to total oxidation. In contrast, partial oxidation catalysts are most often oxides of earlier transition metals, e.g. V and Mo. The suggestion is that the authors clearly state their focus on total oxidation.

Response: Thanks for the comment and suggestion. Indeed, our work in this report focuses on total oxidation of hydrocarbons. In the revision, we have clearly stated this focus in both abstract and manuscript's introduction (See p. 1 and p. 2).

2) The ternary alloy phase diagram of Co-Ni-Pt is never mentioned but is extremely important. What is the miscibility range? Are there any ordered intermetallic phases? The description of the solid structure by a single fcc lattice constant suggests a solid solution, is this known to be the case over the composition range of interest?

Response: Thank you for the comment and question. While phase diagrams for binary PtNi and PtCo are available in the literature, featuring different ordered phases depending on temperature and composition, the phase diagram for ternary PtNiCo is not available. Based on previous studies, this type of nano alloy features a solid solution. For example, in an earlier study of fuel cell catalysts (new Ref 34, Nanoscale 11, 5512-5525 (2019)), XRD/HE-XRD characterizations and Monte Carlo simulation showed that PtNiCo nanoparticles feature a random alloy in a wide range of composition. In the present work, the PtNiCo catalysts prepared with different compositions treated at different temperatures were characterized by XRD and HE-XRD, showing the fcc-type structure characteristic of a solid solution. We also note the fact that almost all available phase diagrams for binary or ternary systems are obtained from bulk materials. Phase diagrams or miscibility properties of nanoscale materials are increasingly known to be significantly different from bulk materials due to the much higher surface area-to-volume ratio, the atomic mobility, and the thermodynamics at the nanoscale. This is a well-known phenomenon now, as exemplified in our earlier work for binary nanoparticles in terms of melting point decrease and miscibility gap removal [e.g., J. Mater. Chem., 21, 4012 (2011)], and supported by theoretical modeling of the phase diagram based on thermodynamics at the nanoscale [e.g., Acc. Chem. Res. 49, 1587 (2016), new Ref. 33]. Given this consideration, the bulk phase diagram, even found, will not have significant value to the structural analysis of the nanoscale ternary system. Part of our future work is to perform theoretical computation/simulation of the nanoscale phase diagrams, which we hope to provide additional insight. In our present work, the phase properties are clearly evidenced by the HE-XRD/PDF analysis (See Supplementary Fig. 5). We clarified this point in the revised manuscript. (p.10 and 11.)

3) Line 95 – "further confirms the presence of the surface sites with Ni and Co atoms in the nearest neighbor of Pt atoms." It's not obvious how the figure tells us this, particularly since TEM sees through the entire particle. Since it is an important point the authors build on later on, it is important to explain how they came to this conclusion.

Response: Thanks for the comment. The Reviewer is correct that the TEM see through the entire particle and cannot tell the presence of surface sites. It should have been about the lattice features. The statement in line 95-96 is thus revised as "further confirms the presence of Ni and Co atoms in the lattice". In our work, we have several pieces of evidence that allowed us to draw this conclusion. First, the HR-TEM shows a randomized lattice spacing and intensity over the entire nanoparticles (see Supplementary Fig. 2); The irregular interatomic distance reflects an intermixing pattern of Pt, Ni, Co, which is also reflected by the uniform composition distribution across the entire nanoparticle in elemental mapping (see Fig.1). Secondly, XPS results show that the surface composition is very close to that of ICP analysis of the nanoparticles. In addition, as shown by Reversed Monte Carlo simulation (New Ref 34, Nanoscale 11,

5512-5525 (2019).), PtNiCo nanoparticles featured the random alloy characteristic. In addition to deleting the phrase line 147-148, we have incorporated the above points into the revised version (p.3. and p. 9).

4) Figure 1 and the discussion surrounding it on page 3 – the surface layer in Fig 1a is not obvious and in the accompanying Fig. S3a the labels on the image were illegible on my copy. The discussion of strain, I don't understand the significance of the strain with respect to Pt, should be with respect to the bulk alloy with the composition of interest, and definitely need to indicate whether that strain is compressive or tensile.

Response: Thanks for pointing out the figure clarity issue and the comments. In the revision, we have improved the quality of the figure image for a better clarity on the illustration of the surface layer. This was done by refining the contrast for better clarity in the HR-TEM image. As for the discussion of strains, we note that the lattice strain of PtNiCo is with respect to Pt and is compressive due to lattice shrinking by alloying. The degree of compressive strain reflects the insertion of oxygen in the lattice with a subtle deviation from Vegard's law depending on the degree of oxidation, which is an important element for controlling surface oxygenation. In the revision, we have clarified this point (p.4.).

5) Also on page 3, concerning the EXAFS analysis, in the fcc structure how do you get >12 nearest neighbors (Ni-Ni = 13.2)? I don't follow how the numbers lead to the suggestion of an ordered-layered structure. An ordered layered structure would not be consistent with a solid solution with an fcc structure. Review of information about what is known about the bulk ternary alloy would be helpful here.

Response: Thanks for the comment and question. The Ni-Ni CN number 13.2 was due to the large uncertainty in fitting the quite noisy Ni spectrum in the EXASF data, which had a standard deviation of 3.4. Since our major interest here is the M-O's CN, we removed this sentence on page 3 to avoid confusion, which did not influence the conclusion. Our initial assessment for "ordered-layered structure" was based on the high coordination numbers of Pt-Pt, Ni-Ni and Co-Co observed in XAFS data. Since part of the fitting was affected by the large uncertainty of the noisy Ni spectrum, we mainly focused on the M-O CNs for the assessment of the degree of surface oxygenation. In the revision, we have made the correction in this context using "surface oxygenated layer", which closely reflects our conclusion (p.4.).

6) Catalyst aging – the aging conditions are very extreme compared to their operating conditions, did the authors see any changes in activity with time on stream?

Response: Thanks for the question. Our use of the extreme aging condition stemmed from the potential application of the catalysts in the harsh conditions of emission control systems. In our work, the activity with time on stream was evaluated under several conditions, and the results are shown in Fig. 3 and Supplementary Figs. 9 and 21, where no significant changes were observed except subtle fluctuations for up to 1 hr stream. Note that the activity depends on the ternary composition during the aging process. The PtNiCo-PtNiOCoO catalysts showed a slight increase in activity, which is higher than that of the Pt catalyst. The Pt catalyst showed a slight decrease under propane oxidation condition (See Supplementary Fig. 21). In the revised version, we have added a comment to reflect this aspect (p. 5 and 7).

7) Figure 3 is so compressed that it is almost impossible to understand, it was only by looking in detail at the supplement that I was able to tell what was going on. The axis on the 3D plots are impossible to see, the color 3D IR spectra do not really show peaks that are easy to see when compared to the text, two of the three insets in the mass spec trace are impossible to see. The point about oscillations in the surface species does not come through at all, it is only in Figs. S19 and 21 that the reader can be convinced that this in fact occurs.

Response: Thanks for the comments. It is an excellent point that we need to improve visualization of Figure 3. In the revised version, we update the figures by decompressing the figure density and improving the visualization. This was achieved by moving the three 3D graphs to the Supplementary Information, i.e., the original graphs c, e and g in Fig. 3 are now moved to Supplementary Fig 6. The

remaining graphs a, b, d, f, and h are now the decompressed, and refined as shown in Fig. 3, showing better clarity and visualization (p.6.).

8) Mass spec data – m/z = 44 corresponds to the parent ions of both CO2 and propane. The authors should try to correct the m/z = 44 trace for the propane contribution by analyzing the cracking pattern, e.g. the 44/29 ratio when only propane is leaked into the mass spectrometer.

Response: Thanks for pointing it out and the comment. In our work, we used the standard 44/29 ratio from the database, and the m/z = 44 trace for the propane contribution was corrected based on the database, which is ~ 22% for better illustrating CO_2 content in Fig. 3. We did not do 44/29 correction because in the mass spectrometer the propane signal was significantly lower than that of CO_2. For this reason, m/z = 44 is specified in the figure.

9) Page 7 Line 228 – "introducing lattice vacancy" where does this conclusion come from?

Response: Thank you for the question. This assessment should have been stated as "introducing lattice oxygen". It was largely based on a combination of activity, HE-XR, XPS, XASF and DRIFT data. In the activity data, there is an indication of lattice oxygen contribution from the oxygenated surface (see added Supplementary Fig. 11); In the XPS and XASF data, significant M-O CNs were detected; In the DRIFT data, surface intermediates removal/growth were observed; In the in-situ XRD/PDF analysis, reversible lattice expansion/shrinking processes were revealed as a result of surface oxygenation and deoxygenation. The lattice expansion/shrinking (Fig. 3) is indicative of the occurrence of lattice vacancy. To further substantiate this conclusion, we have added Supplementary Fig. 11 demonstrating that the lattice oxygen is responsible for the catalytic activity, where the decrease of propane and the increase of CO_2 for the oxygenated ternary alloy catalysts are in sharp contrast to the data for the Pt catalyst. For a better clarity, we have added the description to reflect the above points (p. 7).

10) Page 9 Line 309 – "regular-irregular oscillatory kinetics" This term is used in several places, I don't know what it means.

Response: Thanks for the comment. The term "regular-irregular oscillatory kinetics" stems from both experimental observation and theoretical modeling computation. The experimental observations are from our in-situ/operando characterizations (HE-XRD/PDF, DRIFT, MS, in Fig. 3). The theoretical modeling is from earlier Monte Carlo simulation of the oscillatory kinetics for CO oxidation by Zhdanov (Ref. 30), and several additional refs by the same author (e.g., J. Catal. 214 (2003) 121, Chem. Phys. Letts. 400 (2004) 245, and Catal. Lett. 69 (2000) 21), revealing the size effect on oscillatory behavior for CO oxidation reaction. When size decreases, the oscillatory behavior becomes more irregular especially when the size is smaller than 4 nm. In our case, the nanoparticle size is around 5 nm right at the boundary of the regular and irregular transition region. We have clarified this point in the revised version. (p.9-10).

11) Also on page 9, first time experimental evidence of oscillatory kinetics. As a blanket statement this certainly can't be true. Are they referring specifically to the Monte Carlo calculation referenced? This statement needs to be clarified.

Response: Thanks for the comment and question. The reviewer is correct that we were referring specifically to the Monte Carlo calculation referenced. While oscillatory kinetics is well known, the "regular-irregular oscillatory kinetics" has not been observed experimentally in the literature except simulation work. Referring to our explanations in response to the previous question #10, we believe that the "regular-irregular oscillatory kinetics" captured by our in-situ/operando characterizations has provided for the first time the experimental evidence for the results from earlier Monte Carlo simulation of the oscillatory kinetics. To address this concern, our statement is refined for a better clarification (p.9-10).

Reviewer #3:

As the authors mentioned, oxidation reactions, total or partial oxidations, are widely used in the chemical industry and environmental governance, and the activation of gaseous oxygen is very important for these applications. The multi-component synergy of catalysts is one of the most important research topics in heterogeneous catalysis, and the interfacial interaction between different components is one of the key factors in determining the performance of catalysts. The present work reported an interesting phenomenon that the surface oxygenation of a multicomponent alloy of Pt with oxyphilic metals such as Ni and Co under catalytic reaction condition can generate multifunctional surface-active sites with substrate-active Pt center and surface oxygen-activating NiO/CoO sites. By using in-situ/operando time-resolved characterizations, the authors successfully show reasonable explanation and make fruitful discussion. Some insights in this phenomenon is expected to generate wide interest in this area. I recommend it to publish after a minor revision. Here are some specific comments for consideration during the revision.

Response: We appreciate the Reviewer's overall assessment of the impact of our insights, and considered the specific comments in the revision.

1.The authors called the surface oxygenation of Pt-alloyed multicomponent nanoparticles an Oxidation Catalysts. I cannot agree with it. In general, an oxidation catalyst means a non-metal oxidation catalyst. In the present work, the Pt-alloy is only partially oxidized.

Response: Thanks for the comment. The Reviewer is correct and his/her point on oxidation catalyst means a non-metal oxidation catalyst is well taken from the perspective of traditional oxidation catalysts. In our work, the strategy for the catalyst design involves surface oxygenation of Pt based alloy catalysts which serve as a new oxidation catalyst. Referring to our response to Reviewer 1's question #1, this is a new design strategy for oxidation catalysts because it directly creates the active sites from a pre-alloyed composition with much intimate atomic synergy, rather than the metal oxide supported catalysts where the active sizes are created at the metal/support perimeter zone. To clarify this point, we have incorporated the discussion into the revised version (p.2).

2.Since the active sites are very complicated, the calculation of TOF is questionable. Can the oxidation degree be added as a correction factor? The authors used changes of TOF over different samples to support their concussion that that the catalytic activity originates from the ternary compositions, which needs further discussion and more evidences.

Response: Thanks for the comment and question. We agree the active sites are very complicated. Our TOF calculation was based on the finding from XPS characterization showing that the surface composition remained unchanged even after hydrothermal aging, and was quite similar to the nanoparticle composition. Pt remains on the surface as part of the surface oxygenated layer, and the non-noble metal species function as an oxygen reservoir for oxygen activation. TOF was thus calculated by converting the reaction rate over dispersed Pt species on the surface (Ref. 4). The reaction rate was normalized by Pt. Thus, considering the same surface and bulk composition, i.e., Pt specific dispersion= surface Pt over total available Pt species, we have

TOF = R/(MW(Pt in oxygenated alloy)×D),

where R represents reaction rate and D is the dispersion of Pt on the surface of NP. Based on a cone-shaped volume, D can be calculated by

$D = (6V_m/(a_m\ d))\times 100\%$,

where V_m is the volume of atom Pt, a_m is Pt surface area, and d is the particle diameter. Pt specific reaction rate was normalized to Pt sites on the surface to calculate the TOF. The oxidation degree did not seem to affect the distribution significantly, and thus a correction factor was needed. However, the particle size may change upon surface oxygenation depending on the composition. In this case, TOF was calculated using the d value obtained after the surface oxygenation, showing a subtle increase in TOF value as shown in Supplementary Table 3. The further determination of the effect of oxygenation degree on the detailed surface structure and composition is part of our future work. To address the Reviewer's

concern, we have reflected part of the above description in the revised version (p.8, and Supplementary Table 3).

- *Is it possible that the surface oxidation of metal catalyst is common phenomenon? In the present work, what is the role of the alloy during self-oxidation. What happened for a single metal catalyst during the catalytic process?*

Response: Thanks for the questions. While surface oxidation of metal catalysts is common, the degree of oxidation depends on a combination of factors. The degree of oxidation strongly depends on the alloying composition and phase structures. Referring our response to Reviewer 1's question #1, the role of the alloy during self-oxidation is the change in oxidation potential since resistance to oxidation or the formation passivation layer depend on the chemical nature of the alloying composition and structure. For single metal Pt catalyst (Ref. 24), the surface oxidation would form PtOx leading to possible surface poisoning and sublimation of PtOx. In the case of PtNiCo, Ni and Co are more prone to oxygenation than Pt, and serve as both oxidation-sacrificing metals and lattice oxygen sites on the surface, leading to minimization of Pt oxidation and suppression of surface poisoning. This is partially evidenced by our XASF data, revealing much smaller Pt-O coordination numbers in the PtNiCo alloy than those in the PtNi and PtCo catalysts. We have clarified these points in the revised version (p.3).

3.The authors highlighted that the self-activated catalysts are stable oxidation catalysts. However, no related data is given to show what will happen if the used catalysts are pretreated in reduction atmosphere for reuse.

Response: Thanks for the comment. In the manuscript, we compared the catalytic activity data between oxidatively and reductively treated catalysts: upon the reduction, the catalytic activity was shown to drop slightly, indicating the importance of surface oxygenation. (See Supplementary Fig. 14B.).

4.The discussions on reaction mechanism is a little confusing, especially on the Langmuir–Hinshelwood or Mars–van Krevelen pathway over different sites.

Response: Thanks for the comment. We should have stated clearly the Mars–van Krevelen pathway since we believe the reaction mechanism follows MK pathway mainly because of several reasons. First, the observation of the oscillatory behavior of surface intermediates and structure indicates that the surface oxygen plays an important role in the reaction. Second, as shown in the in-situ MS data by the oxygen spike at the beginning of the reaction (Fig. 3), oxygen was less consumed whereas propane consumption was growing, indicating that propane reacts with surface oxygen in oxygenated layers. Third, the surface oxygen is found to be very active in propane oxidation. This was evidenced in evaluating the catalytic activity by purging propane feeding gas only (without O_2) through the oxygenated catalysts. The activity data were then monitored and compared between PtNiCo catalyst and Pt catalysts under N_2 protected atmosphere. As shown by the result (See added Supplementary Fig. 11), there are clear activities showing the decrease of propane and the increase of CO_2 for the oxygenated ternary alloy catalysts, in sharp contrast to the data for the Pt catalyst. The result demonstrate that the surface oxygen is the leading active species in catalytic reaction, as expected by the MK mechanism. In the revised version, we have refined the description on reaction mechanism and incorporated the above discussion and data (p.7 and p.10).

We thank again all three reviewers for their thoughtful, insightful, and helpful comments, questions, and suggestions.

REVIEWERS' COMMENTS:

Reviewer #2 (Remarks to the Author):

The authors have addressed my comments, the paper can now be published pending one comment. The axis labels in the plots in Figs. 1, 3 and 4 are microscopic, please use a larger font.

Reviewer #3 (Remarks to the Author):

The manuscript is satisfied for acceptance after the revision.

Point-by-point responses and revisions made in the manuscript (Manuscript ID: NCOMMS-20-17136A)

Reviewer #2:
The authors have addressed my comments, the paper can now be published pending one comment. The axis labels in the plots in Figs. 1, 3 and 4 are microscopic, please use a larger font.

Response: Thanks for the suggestion, we have enlarged the fonts in the axis labels of the plots for Figs. 1, 3 and 4. We have also done so for other figures or plots as well.

Reviewer #3:
The manuscript is satisfied for acceptance after the revision.

Response: No comment for us to respond.
